# PUF family RNA-binding proteins Puf1 and Puf2 promote transcript degradation in *Toxoplasma gondii*

**DOI:** 10.1016/j.jbc.2026.113436

**Published:** 2026-08-12

**Authors:** Chitti Raju Khandavalli, Rajkumar Gurupwar, Abhijit S. Deshmukh

**Affiliations:** 1 Molecular Parasitology Laboratory, BRIC-National Institute of Animal Biotechnology, Hyderabad, Telangana, India; 2 Regional Centre for Biotechnology (RCB), Faridabad, Haryana, India

**Keywords:** apicomplexan, *Toxoplasma gondii*, PUF, RNA-binding protein, RNA degradation, deadenylase

## Abstract

*Toxoplasma gondii*, a highly successful apicomplexan parasite, primarily relies on post-transcriptional mechanisms to regulate mRNA stability and translation during rapid life-cycle stage transitions and to adapt to diverse host environments. While RNA-binding proteins (RBPs) are crucial for these regulatory processes, their specific roles in mRNA translation, storage, and degradation during life-stage transitions and under physiological stress in *Toxoplasma* remain poorly understood. Here, we identified the PUF family of RBPs and characterized two conserved members, TgPuf1 and TgPuf2. We examined their expression, localization, RNA-binding activity, essentiality during asexual stages in cell culture and mouse host, responses to stress conditions, and roles in transcript regulation. Gene-knockout studies in cell culture showed that TgPuf1 modestly supports parasite fitness under both normal and stress conditions, while TgPuf2 appears largely dispensable. Mice infected with Puf1-deleted tachyzoites showed delayed mortality compared with wild-type, whereas neither Puf1 nor Puf2 deletion affected bradyzoite development. Both TgPuf proteins bind to a conserved RNA sequence known as PUF recognition elements (PREs), associate with ribonucleoprotein complexes, and interact with the deadenylase enzyme TgPop2. Using reporter gene systems, we demonstrated that, upon interaction with TgPop2, TgPuf proteins stimulate the removal of the poly(A) tail from RNA targets, thereby promoting RNA degradation. The inability to generate the double knockout is adequately addressed using TgPuf1-mAID in the delta TgPuf2 background, indicating that individual Puf proteins are dispensable; however, the lack of both results in severe growth defects. Overall, these findings suggest that PUF proteins regulate transcript levels in *Toxoplasma* through a deadenylation-dependent mechanism.

A member of the phylum *Apicomplexa*, *Toxoplasma gondii* is an obligate parasite that causes the disease toxoplasmosis in warm-blooded animals, including humans (1, 2). The infection can lead to severe, potentially life-threatening disease in fetuses and immunocompromised individuals (2, 3). The heteroxenous life cycle of *Toxoplasma* involves sexual reproduction in feline definitive hosts and asexual reproduction in other intermediate hosts with three infective stages: (1) tachyzoites, rapidly multiplying, causing acute infection; (2) bradyzoites, slowly growing within tissue cysts; and (3) sporulated oocysts, environmental forms shed in the feces of the feline host (3, 4, 5). Throughout its complex life cycle, the parasite rapidly adapts to diverse pressures from both intracellular and extracellular environments, as well as different host tissue types and species, involving significant changes in gene expression profiles (6, 7, 8, 9). Emerging evidence suggests that both transcriptional and translational regulations play critical roles in controlling gene expression during development and adaptation to environmental changes; however, the underlying mechanisms remain largely unexplored (6, 8).

Translational regulation of gene expression is crucial in most organisms, as it enables the cell to respond more rapidly to external stimuli than transcriptional regulation (6, 8). In metazoans, translational control is significant during early development, when the developing embryo relies on stored maternal mRNAs that become activated for translation after fertilization (10). Similarly, in apicomplexan parasites like *Toxoplasma* (6, 11, 12, 13, 14, 15) and *Plasmodium* (16, 17, 18), translational regulation plays a crucial role in modulating stage conversion and host–parasite interactions by repressing the translation of stage-specific transcripts. In *Plasmodium* spp., translational repression is prominent in female gametocytes, affecting various mRNAs that encode for surface proteins, regulators, and proteases (16, 19, 20, 21). For example, in *Plasmodium berghei*, *Pbs21* transcripts, a surface antigen, are found in female gametocytes; however, the translation of *Pbs21* mRNA occurs only when the parasite enters the mosquito gut (22, 23). In *Toxoplasma*, the mRNA of the BFD1 protein, the master regulator of chronic bradyzoite differentiation, is present in acute-stage tachyzoites but is translated exclusively under bradyzoite-inducing stress, indicating its expression is mainly regulated through translational control (12, 24).

Translation control in metazoans can occur through the interaction of RNA-binding proteins (RBPs) with cis-acting sequence elements, which are typically located in the 5′ and 3′ UTRs of mRNAs (25, 26, 27). This interaction regulates mRNA stability, localization, and translational efficiency (28, 29). A notable example of RBPs is the PUF proteins (Pumilio and fem-3 binding factor), characterized by the Pumilio homology domain, a conserved RNA-binding domain (30, 31, 32, 33). The PUF family is a large group of RBPs found in all eukaryotes, with the number of Puf genes varying from 2 to 26 across different model organisms (34, 35). Within the PUF family, three types of proteins have been identified: Puf, Nop9, and PUM3, which are distinguished based on their biological roles and structural arrangements (34, 35). A classical Puf protein contains a Pumilio homology domain (PUM-HD), which is formed from eight α-helical (Puf) repeats, each of ∼38 amino acids long (36, 37, 38). This domain binds to a single base within a target RNA sequence, enabling the entire domain to interact with an eight-base RNA strand (UGUA[N]AUA, N = any base) known as Puf recognition elements (PREs) in target mRNAs (39, 40, 41, 42). Puf proteins regulate gene expression by binding to PREs in target mRNAs and recruiting the Ccr4-Pop2-Not deadenylase complex to remove the poly(A) tail, promoting mRNA decay and translational repression (43).

The significance of Puf proteins in several key cellular functions is well-documented in metazoans and model organisms; however, their functions in protozoan parasites, particularly apicomplexans, remain poorly understood. Among apicomplexan parasites, the role of Puf proteins has been relatively well studied in *Plasmodium* spp. (44, 45). Of the three identified Puf proteins in *Plasmodium falciparum*, Puf1 (46) and Puf2 (45, 47, 48, 49) are classical cytoplasmic proteins essential for gametocytogenesis, while Puf3 is a nuclear protein involved in ribosomal biogenesis (50). In *T*. *gondii*, only the Puf1 protein has been studied for its RNA-binding activity using sequences from different organisms (51). This underscores a significant gap in our understanding of the number, functions, essential roles, and regulatory mechanisms of Puf proteins in *Toxoplasma*.

In this study, we identified the PUF family of RNA-binding proteins and functionally characterized two proteins, TgPuf1 and TgPuf2, during the asexual stages of *Toxoplasma*. TgPuf1 supports parasite fitness and is critical for maintaining parasite viability under physiological stress, while TgPuf2 is dispensable. Both proteins showed similar cytoplasmic localization and binding affinities for RNAs containing PRE, with the double mutant potentially lethal to the parasite, indicating possible TgPuf compensation/cooperation.

## Results

### *T. gondii* encodes four Puf proteins

**Figure 1:**
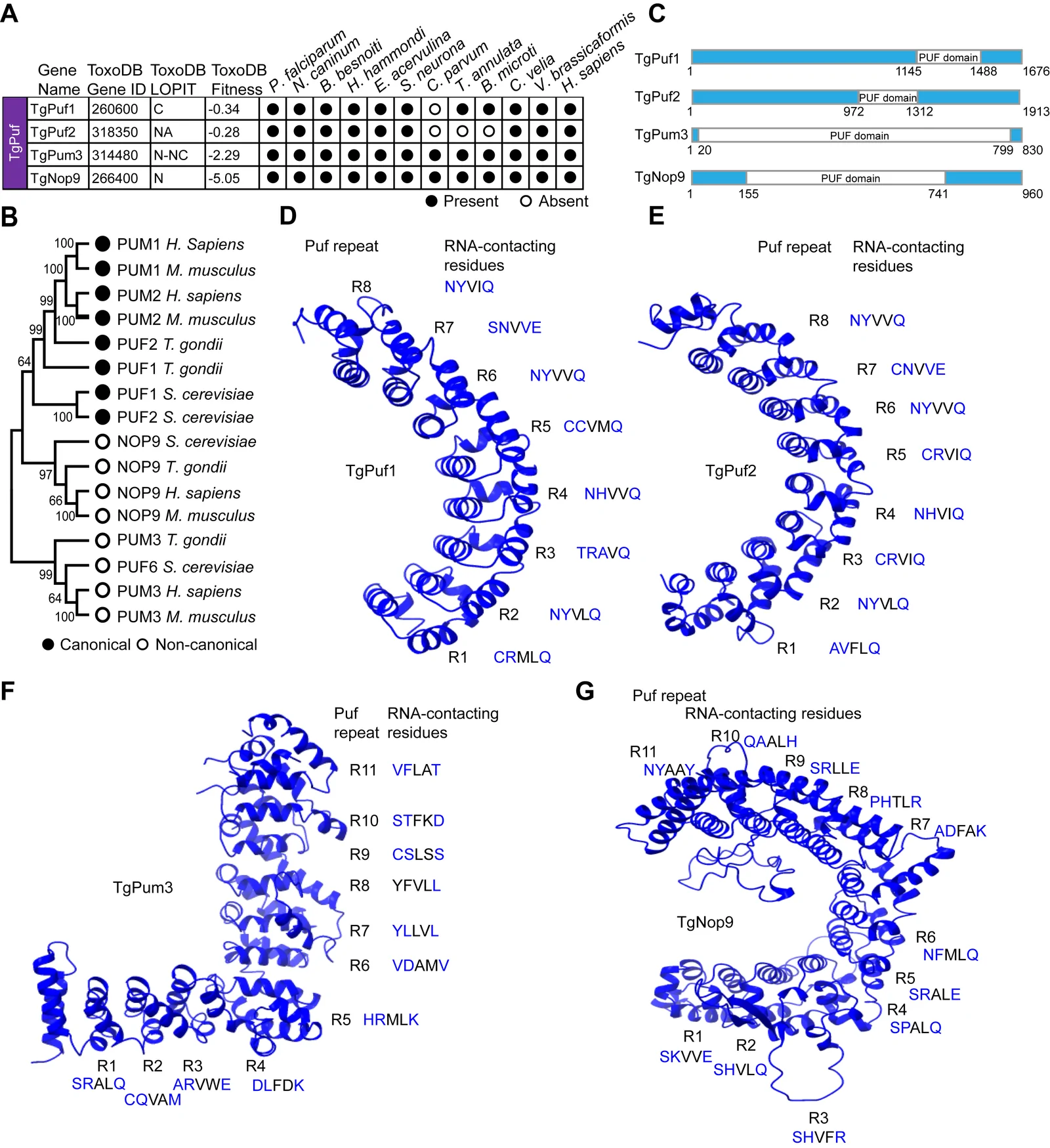
**Overview of Puf family proteins in *Toxoplasma*.***A*, the conservation of Puf protein across the apicomplexans was determined by BLASTp search on VEuPathDB using protein sequences of *H*. *sapiens* and *S*. *cerevisiae*. Summary of Puf proteins, implying localization based on hyperLOPIT data (108) and fitness score derived from genome-wide CRISPR fitness screen (80) in *T*. *gondii*. *B*, phylogenetic tree of Puf proteins (TgPuf1, TgPuf2, TgPum3, and TgNop9) was constructed using MEGA analysis (109) after alignment of full-length protein sequences in 1000 bootstrap replicates. Filled circles represent canonical Puf proteins, while open circles denote non-canonical Puf proteins. *C*, schematic depicting conserved Puf-RNA binding domain in Puf family proteins; TgPuf1 (1145–1488 aa), TgPuf2 (972–1312 aa), TgPum3 (20–799 aa), TgNop9 (155–741 aa). *D–G*, predicted structures of TgPuf1 (1145–1488 aa), TgPuf2 (972–1312 aa), TgPum3 (20–799 aa), TgNop9 (155–741 aa) using ColabFold v1.5.5: AlphaFold2 (110). Number of repeats and RNA-contacting residues for each Puf protein are mentioned.

Puf proteins are found in most eukaryotic organisms, although the number of Puf genes varies significantly among them. To identify the complete repertoire of Puf proteins in apicomplexans, we performed BLASTp homology searches of eleven apicomplexan genomes (52) (https://veupathdb.org/veupathdb/app/) using the amino acid sequences of Puf proteins from *Saccharomyces cerevisiae* and *H*. *sapiens* as queries. This analysis revealed four Puf proteins: Puf1, Puf2, Pum3, and Nop9 in most of the apicomplexans, including two photosynthetic ancestors, *C. velia* and *V. brassicaformis*, except *Theileria* *annulata* and *B*. *microti* (lack Puf2), and *C*. *parvum* (missing both Puf1 and Puf2) (Figs. 1*A* and S1, *A*, *B*). *T*. *gondii* has four Puf proteins (53): two previously identified (51), TgPuf1 (TgME49_260600) and TgPuf2 (TgME49_318350), and two new genes, designated as TgPum3 (TgME49_314480) and TgNop9 (TgME49_266400) (Figs. 1*A* and S2, *A* and *B*). Phylogenetic analysis of *Toxoplasma* Puf proteins showed that these proteins can be divided into two distinct categories: canonical, which includes TgPuf1 and TgPuf2, and non-canonical, consisting of the newly identified TgPuf3 and TgNop9 (Fig. 1*B*). TgPuf1 is a 1676-aa protein with a Puf domain located at the C-terminus (1145–1488 aa), while TgPuf2 is slightly larger 1913-aa protein, with its Puf domain located in the centre (972–1312 aa) (Fig. 1*C*). TgPum3 (830-aa protein) and TgNop9 (960-aa) are relatively smaller proteins, both featuring a central Puf domain (Fig. 1*C*). TgPuf proteins show limited similarity, with the highest homology in the Puf domains. Domain prediction based on amino acid sequences revealed that TgPuf1 (Fig. 1*D*) and TgPuf2 (Fig. 1*E*) each contain eight Puf repeats, while TgPum3 (Fig. 1*F*) and TgNop9 (Fig. 1*G*) contain eleven repeats. Further protein structure analysis using AlphaFold2 revealed that TgPuf1 (Fig. 1*D*) and TgPuf2 (Fig. 1*E*), each containing eight repeat motifs, form a characteristic crescent-shaped structure, consistent with their identification as classical Puf proteins. In contrast, TgPum3 (Fig. 1*F*) and TgNop9 (Fig. 1*G*), both of which have eleven repeat motifs, adopt L-shaped and C-shaped structures, respectively. Overall, *T*. *gondii* has four identified Puf proteins, two canonical and two non-canonical, and further studies were performed only on Puf1 and Puf2 proteins.

### Both TgPuf1 and TgPuf2 proteins are expressed during asexual stages and bind to specific RNA sequences

**Figure 2:**
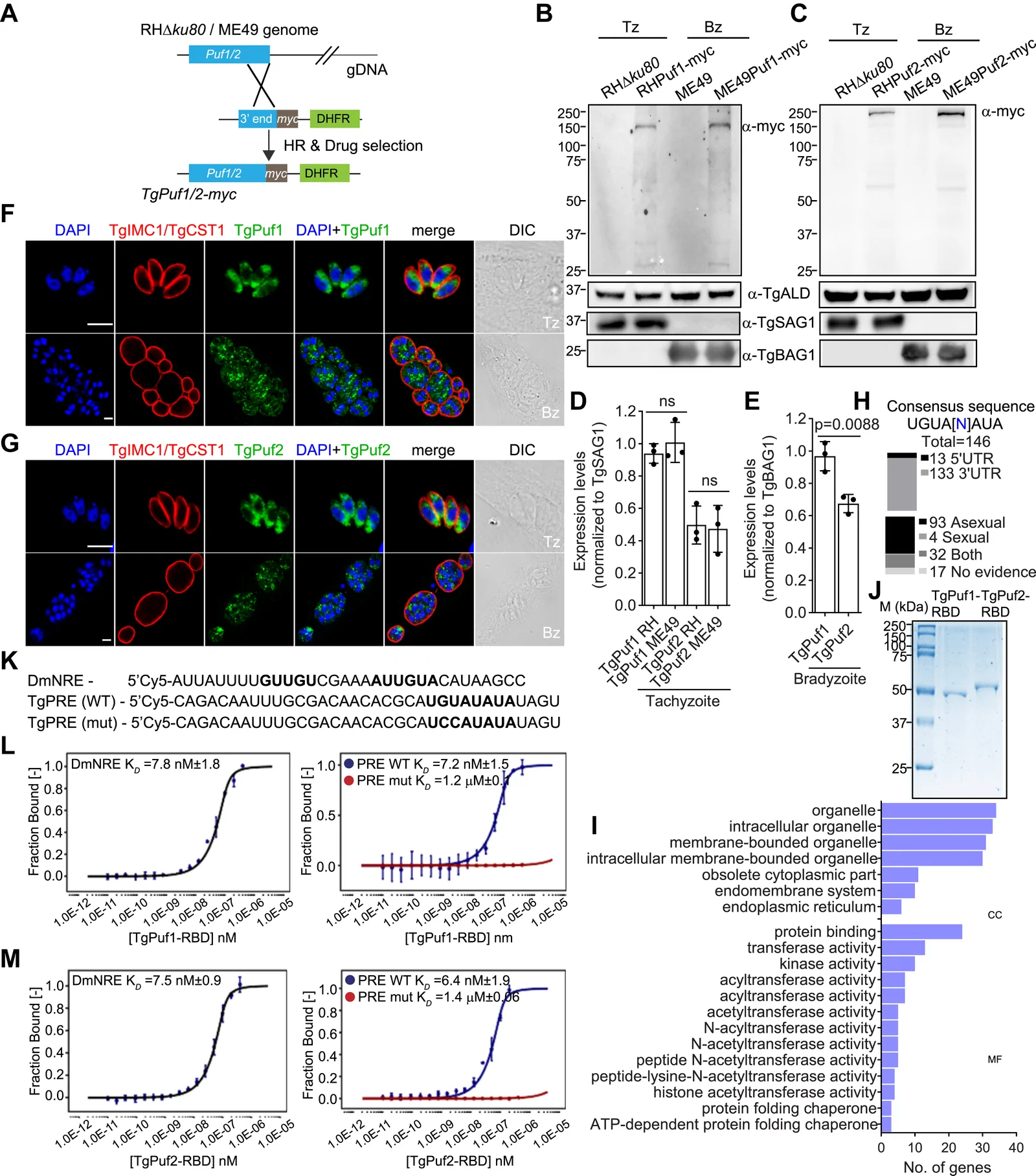
**Both canonical TgPuf proteins show sequence-specific RNA binding.***A*, strategy for C-terminal tagging of TgPuf1 and TgPuf2 protein with a 3x myc tag in RHΔ*ku80* and ME49 strains. *B*, *C*, Expression levels of TgPuf1 (*B*) and TgPuf2 (*C*) in tachyzoites and bradyzoites were assessed using α-myc antibodies. Loading controls: TgAldolase (TgALD)—parasite proteins, TgSAG1—tachyzoite stage marker, and TgBAG1—bradyzoite stage marker. *D*, *E*, Expression levels of TgPuf1 and TgPuf2 in tachyzoites (RH and ME49) and bradyzoites (ME49) normalized to TgSAG1, and TgBAG1, respectively. *F* and *G*, TgPuf1 (*F*) and TgPuf2 (*G*) localization in tachyzoite and bradyzoite stages was analyzed by immunofluorescence using α-myc antibodies. α-TgIMC1 antibody - tachyzoite inner membrane, and α-TgCST1 antibody-bradyzoite cyst wall. Scale bar, 5 μm. *H*, Conserved Pumilio recognition element (PRE) sequence 'UGUA[N]AUA'. PRE-containing genes identified using Bioconductor packages GenomicRanges and Biostrings and their distribution in the different stages of the parasite. *I*, GO analysis of PRE-containing transcripts, focusing on cellular components and molecular functions. *J*, purification profile of TgPuf1 RBD-His (∼53 kDa) & TgPuf2 RBD-His (∼55 kDa) analyzed on 10% SDS-PAGE. *K*, 5′ cyanine labeled DmNRE, TgPRE (WT), and TgPRE (mut) RNA sequences. *L*, *M*, MST analysis of 5′ cyanine labeled DmNRE, TgPRE (WT), and TgPRE (mut) with TgPuf1 (*L*) and TgPuf2 (*M*) proteins.

To first gain insight into the expression and localization of TgPuf proteins (TgPuf1 and TgPuf2) in both asexual stages (tachyzoite and bradyzoite), we generated 3x-myc-tagged lines of these Puf proteins in both the RH and ME49 background strains (Fig. 2*A*, Table S1). Our studies revealed that both proteins are expressed during the asexual stages, with TgPuf1 migrating at 170 kDa (Fig. 2*B*) and TgPuf2 migrating at 210 kDa (Fig. 2*C*), as expected. We also checked the relative abundance of TgPuf1 and TgPuf2 proteins across stages and found that TgPuf1 was expressed more in both tachyzoites (Figs. 2*D* and S3, *A*, *B*) and bradyzoites (Figs. 2*E* and S3, *C*, *D*) than TgPuf2, irrespective of strain (RH or ME49). Furthermore, localization studies using immunofluorescence staining demonstrated that both Puf proteins are located in the cytoplasm of the parasite in both tachyzoite (Fig. 2*F*) and bradyzoite (Fig. 2*G*) stages.

Puf proteins bind to a core consensus sequence 'UGUA[N]AUA' (where N can be A, U, C, or G) known as PRE (Fig. 2*H*). However, the frequency of this sequence in *Toxoplasma* transcripts is still unknown. Using the *Toxoplasma* database, we extracted 5′ untranslated regions (5′UTRs) and 3′ untranslated regions (3′UTRs) from the ME49 genome version 68, yielding 7939 5′UTRs and 7497 3′UTRs. We then screened these regions for the canonical PRE motif. Our initial motif scan identified 834 UTRs containing PRE motifs (from both 5′ and 3′ UTRs). After performing background modeling and correcting for false discovery rate (FDR < 0.05), we identified 146 high-confidence genes that include a PRE motif in their UTRs (133 genes with 3′UTR and 13 genes with 5′UTR) (Table S2). The occurrence of PREs in actual UTRs was significantly higher than in shuffled UTRs (empirical *p* = 0.0099), confirming that PREs are more abundant than expected by chance. Notably, PREs were predominantly found in 3′UTRs (91%), which aligns with the expected regulatory role of Puf family proteins, while a smaller proportion was identified in 5′UTRs.

Next, we examined the expression status of all 146 genes using mass spectrometry-based expression data from ToxoDB (53). Most of these genes (n = 93) were expressed during the asexual stages, while only a few (n = 4) were expressed in the sexual stages. A significant number (n = 32) were expressed in both stages, and expression data for 17 genes were unavailable. Furthermore, gene ontology analysis for cellular components and molecular functions was performed using the ToxoDB GO enrichment tool (53), with *p* < 0.05 for the identified categories. While the cellular component analysis revealed that most of these genes are associated with membrane-bound organelles, the molecular function analysis indicated that they are involved in kinase activity and protein folding (Fig. 2*I*).

To determine the RNA-binding activity of TgPuf proteins, we first bacterially expressed (Table S1) the recombinant RNA-binding domain (RBD) of TgPuf1 (TgPuf1-RBD, 1145–1488-aa) and TgPuf2 (TgPuf2-RBD, 972–1312-aa), as full-length proteins are quite large. Both recombinant proteins showed molecular weights of 53 kDa for TgPuf1-RBD and 55 kDa for TgPuf2-RBD (Fig. 2*J*), as expected. The RNA-binding activity of these recombinant proteins was tested using Cy5-labeled RNA oligos: 1. DmNRE (*Drosophila* hunchback NRE mRNA), 2. TgPRE (WT) (*Toxoplasma* gene with consensus PRE sequence: TgCDPK7A TgME49_237860, selected randomly), 3. TgPRE (mut) (Fig. 2*K*, Table S1 and S2) by MST. For TgPRE (mut), we introduced the two-point mutations (UGUAUAUA to UCCAUAUA) that specifically alter the second and third nucleotides. This minimal substitution is to directly test the sequence specificity without introducing changes in length and GC content, and the secondary structures that would occur in the scrambled sequence.

The results clearly demonstrated that both Puf proteins exhibit high binding affinity to DmNRE (K_*D*_ ∼ 7.5 nM) and TgPRE (WT) (K_*D*_ ∼ 7 nM), while no binding was observed for TgPRE (mut) (Fig. 2, *L* and *M*). These results collectively demonstrate that the consensus sequence in mRNA target genes is essential for Puf protein binding, and this sequence is widely present in the *Toxoplasma* transcriptome.

### TgPuf1 moderately contributes to tachyzoite fitness, while TgPuf2 is dispensable

**Figure 3:**
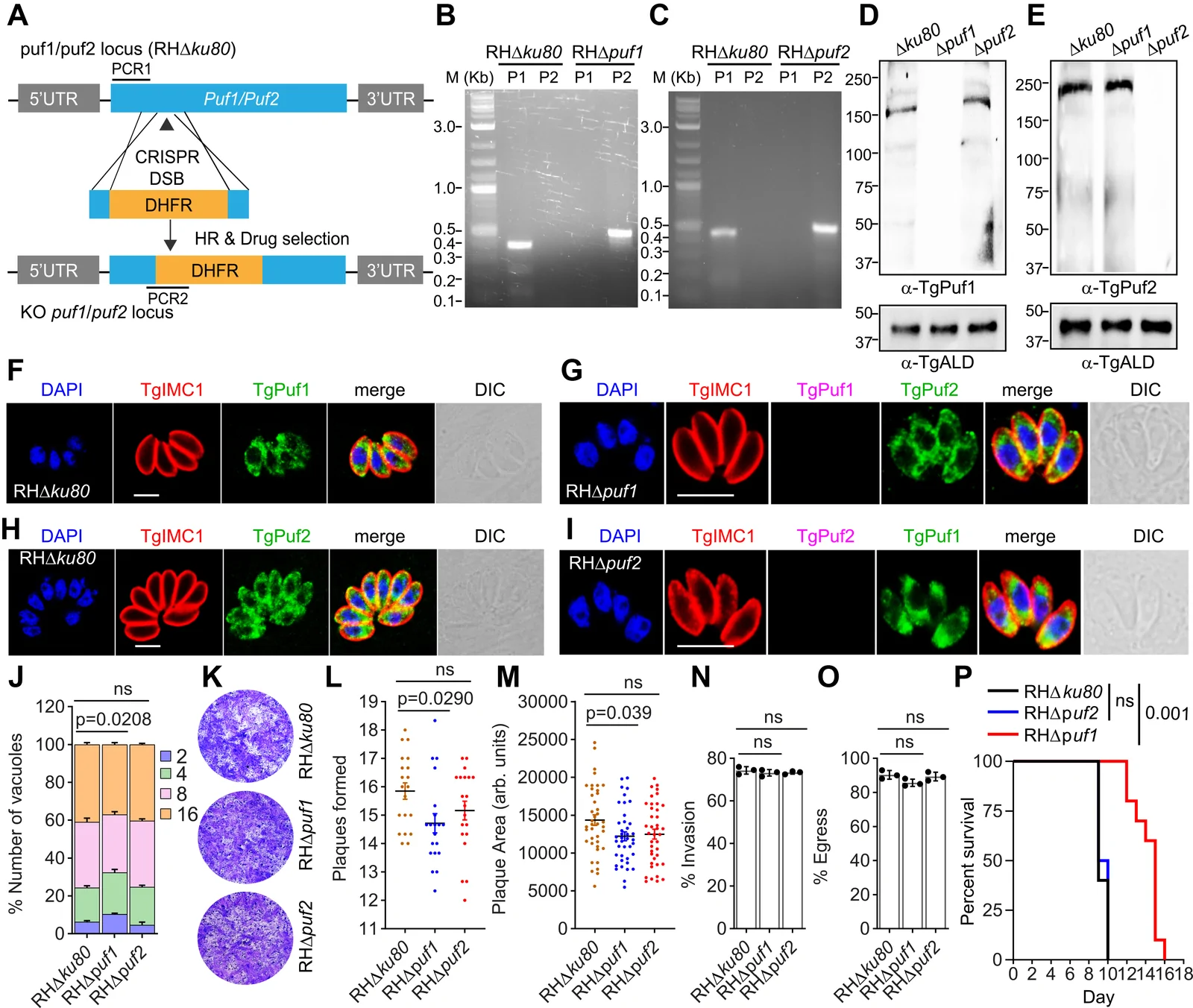
**Effect of TgPuf1 and TgPuf2 deletion on tachyzoite growth.***A*, strategy for TgPuf1 and TgPuf2 knockout in RH*Δku80* parasites. *B* and *C*, diagnostic PCR of genomic DNA from RHΔ*ku80,* RHΔ*puf1* (*B*), and RHΔ*puf2* (*C*) parasites to verify 5′ integration of DHFR at the gene locus. *D* and *E*, expression levels of TgPuf1 (*D*) and TgPuf2 (*E*) in tachyzoites of RHΔ*ku80,* RHΔ*puf1*, or RHΔ*puf2* strains, determined by immunoblotting using α-TgPuf1 or TgPuf2 antibodies. TgALD loading control for parasite proteins. *F*–*I*, Localization of TgPuf1 and TgPuf2 in tachyzoites of RHΔ*ku80,* RHΔ*puf1*, or RHΔ*puf2* strains, tested by immunofluorescence using α-TgPuf1 or TgPuf2 antibodies. Scale bar, 5 μm. *J*, Comparison of parasite replication between RHΔ*ku80* and RHΔ*puf1*, or RHΔ*puf2* strains, determined by immunofluorescence using α-TgIMC1 antibodies. *K*, Crystal violet-stained images of plaques formed by RHΔ*ku80,* RHΔ*puf1*, or RHΔ*puf2* strains on HFF monolayer. *L* and *M*, quantification of plaque numbers (*L*) and plaque areas (*M*) assessed using plaques, as mentioned in '*K*'. *N*, parasite invasion. The graph shows the percent invasion of RHΔ*ku80,* RHΔ*puf1*, or RHΔ*puf2* strains on HFF monolayer. *O*, parasite egress. The graph shows the percent egress of RHΔ*ku80,* RHΔ*puf1*, or RHΔ*puf2* strains after calcium ionophore treatment. Intact or collapsed vacuoles were visualized and counted by IFA analysis. *P*, survival curve of BALB/c mice infected intraperitoneally with 100 tachyzoites of RHΔ*ku80,* RHΔ*puf1*, or RHΔ*puf2* strains. The Gehan-Breslow- Wilcoxon test was used to compare differences between the survival curves. Fig. *L*–*P*; n = 3 replicates; mean ± S.D; one-way ANOVA; Dunnett’s comparison test; ns not significant; *p* values: ∗ <0.05; ∗∗∗ <0.001; ∗∗∗∗ <0.0001.

To test the essentiality of TgPufs for tachyzoite growth, we created knockout strains of TgPufs (RHΔ*puf1* and RHΔ*puf2*) by introducing a dihydrofolate reductase (DHFR) cassette that confers pyrimethamine resistance into the ORF of the TgPuf genes, downstream of the ATG start codon (138 bp for TgPuf1 and 90 bp for TgPuf2), using the CRISPR-Cas9 system (Fig. 3*A*, Table S1). The successful insertion of the DHFR cassette and the absence of Puf protein expression in the knockout strains were confirmed through diagnostic PCR (Fig. 3, *B* and *C*, Table S1), immunoblotting (Fig. 3, *D* and *E*), and immunofluorescence staining (Fig. 3, *F*–*I*). The expression levels and localization pattern of TgPuf1 in RHΔ*puf2* parasites (Fig. 3, *D* and *I*, Fig. S4) were similar to those observed in the parental strain, and *vice versa* (Figs. 3, *E*, *G* and S4). Next, we analyzed the impact of TgPuf1 and TgPuf2 deletion on the lytic cycle of the parasite. The knockout of TgPuf1 resulted in a marginal defect in parasite replication, while the deletion of TgPuf2 had no effect on replication (Fig. 3*J*). The same has been reflected in the parasite growth assay, where knockout of TgPuf1 showed slightly fewer plaques compared to the parental strain (Fig. 3, *K* and *L*). In contrast, plaque numbers were not different in TgPuf2 knockout parasites compared to the parental RH strain (Fig. 3, *K* and *L*). A similar trend was observed for plaque area (Fig. 3*M*), aligning with the results for plaque numbers. We further examined how the deletion of TgPufs affects two crucial processes in parasites: invasion and egress. Knockout of either TgPuf1 or TgPuf2 had no effect on parasite invasion (Fig. 3*N*) and egress (Fig. 3*O*). Finally, to test the effect of TgPuf1 and TgPuf2 deletion on parasite virulence, we injected mice with a lethal dose of either parental or knockout parasites and monitored them for mortality. Mice injected with either the parental RH strain or the RHΔ*puf2* parasites experienced 100% mortality by day 11 (Fig. 3*P*), while those injected with RHΔ*puf1* parasites displayed delayed mortality, with all mice succumbing by day 16 (Fig. 3*P*). Together, these results indicate that the TgPuf1 contributes moderately to parasite growth *in vitro* and *in vivo*, whereas TgPuf2 is dispensable for the tachyzoite stage of *Toxoplasma*.

### Both Puf proteins are dispensable for the bradyzoite stage

**Figure 4:**
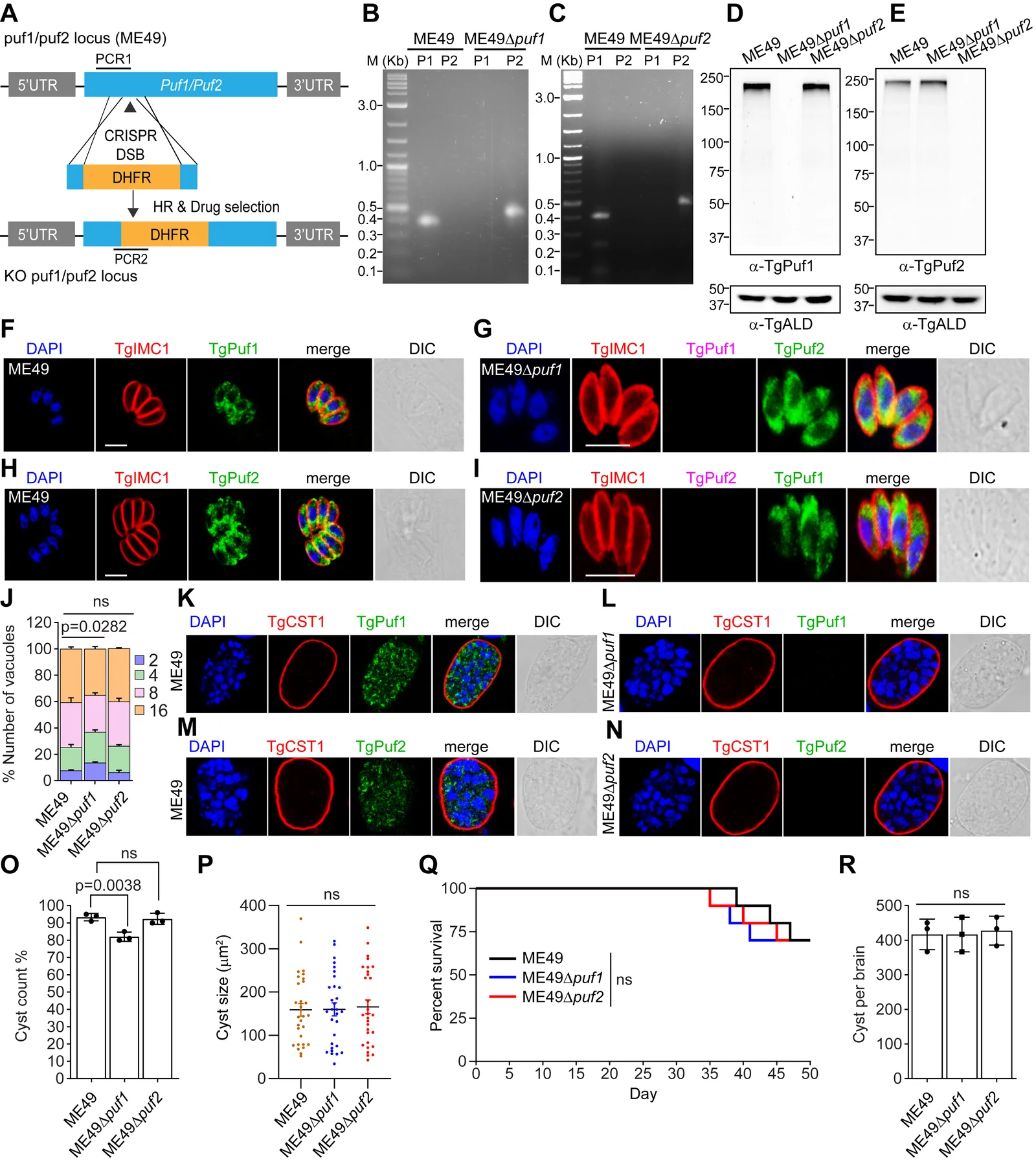
**Effect of TgPuf1 and TgPuf2 deletion on bradyzoite growth.***A*, strategy for TgPuf1 and TgPuf2 knockout in ME49 parasites. *B* and *C*, diagnostic PCR of genomic DNA from ME49, ME49Δ*puf1* (*B*), and ME49Δ*puf2* (*C*) parasites to verify 5′ integration of DHFR at the gene locus. *D* and *E*, expression levels of TgPuf1 (*D*) and TgPuf2 (*E*) in tachyzoites of ME49, ME49Δ*puf1*, or ME49Δ*puf2* strains, determined by immunoblotting using α-TgPuf1 or TgPuf2 antibodies. TgALD loading control for parasite proteins. *F–I*, Localization of TgPuf1 and TgPuf2 in tachyzoites of ME49, ME49Δ*puf1*, or ME49Δ*puf2* strains, tested by immunofluorescence using α-TgPuf1 or TgPuf2 antibodies. Scale bar, 5 μm. *J*, Comparison of parasite replication between ME49 and ME49Δ*puf1,* or ME49Δ*puf2* strains, determined by immunofluorescence using α-TgIMC1 antibodies. n = 3 replicates; mean ± S.D; one-way ANOVA; Dunnett’s comparison test; ns not significant; *p* values: ∗ <0.05; ∗∗∗ <0.001; ∗∗∗∗ <0.0001. *K-N*, Bradyzoite stage immunofluorescence analysis of ME49, ME49Δ*puf1* (*K*, *L*), or ME49Δ*puf2* (*M*, *N*) strains using α-TgPuf1 or α-TgPuf2 antibodies. α-TgCST1 antibody was used to stain the Bradyzoite cyst wall. Scale bar, 5 μm. *O*, *P*, Quantification of cysts formed (*O*) and cyst size (*P*) by ME49, ME49Δ*puf1*, or ME49Δ*puf2* strains. Cysts were identified by staining of the cyst wall by α-TgCST1 antibodies. *Q*, Survival curve of BALB/c mice infected with 500 tachyzoites of ME49, ME49Δ*puf1*, or ME49Δ*puf2* strains, analyzed using the Gehan-Breslow-Wilcoxon test. *R*, Quantitative assessment of the cyst load in the brains of BALB/c mice infected with ME49, ME49Δ*puf1*, or ME49Δ*puf2* parasites, sacrificed on day 50 post-infection. Fig *O*, *P*, *R*; n = 3 replicates; mean ± S.D; two-way ANOVA with Tukey’s *post hoc* test; ns not significant; *p* values: ∗ <0.05; ∗∗∗ <0.001; ∗∗∗∗ <0.0001.

The essentiality of TgPufs for bradyzoite growth was examined by generating knockouts of TgPuf proteins (ME49Δ*puf1* and ME49Δ*puf2*) in the ME49 background strain. We utilized a similar approach as with the RH strain, inserting the DHFR cassette into the ORF of the TgPuf genes, located downstream of the ATG start codon, using the CRISPR-Cas9 system (Fig. 4*A*, Table S1). The successful insertion of the DHFR cassette and the lack of Puf protein expression in the knockout strains were confirmed through diagnostic PCR (Fig. 4, *B* and *C*, Table S1), immunoblotting (Fig. 4, *D* and E), and immunofluorescence staining (Fig. 4, *F–I*), respectively. The expression levels and localization pattern of TgPuf1 in ME49Δ*puf2* parasites (Figs. 4, *D*, *I* and S5) were similar to those observed in the parental strain, and *vice versa* (Figs. 4, *E*, *G* and S5). Subsequently, we analyzed the impact of TgPuf1 and TgPuf2 deletion on parasite replication. The knockout of TgPuf1 led to a slight defect in parasite replication, whereas the deletion of TgPuf2 showed no effect on replication (Fig. 4*J*). Further, we examined the ability of TgPuf knockout parasites to form bradyzoites *in vitro* by inducing stage differentiation using a standard alkaline stress method. Both ME49Δ*puf1* (Fig. 4, *K* and *L*) and ME49Δ*puf2* (Fig. 4, *M* and *N*) parasites were able to form proper bradyzoites, similar to the parental ME49 strain. However, the total number of cysts formed was slightly lower in the TgPuf1 knockout parasites, while the number was not significantly different in TgPuf2 knockout parasites compared to the parental ME49 strain (Fig. 4, *O* and *P*). Finally, to test the effect of TgPuf1 and TgPuf2 deletion on bradyzoite formation *in vivo*, we injected mice with 500 tachyzoites of either parental or knockout parasites and monitored them for 50 days. Three mice from each group (parental or knockout) died between days 35 and 50 (Fig. 4*Q*). After 50 dpi, all surviving mice were sacrificed, and their brains were harvested and examined for cysts using TgCST1 immunofluorescent staining. Cyst quantification data revealed that the number of cysts formed in mice infected with the knockout strain (ME49Δ*puf1*/*2*) was similar to that of the mice infected with the parental ME49 strain (Fig. 4*R*). Overall, these results indicate that neither TgPuf protein is essential for the growth of the bradyzoite stage in *Toxoplasma*.

### TgPuf proteins exhibit punctate staining and form RNP complexes under stress

**Figure 5:**
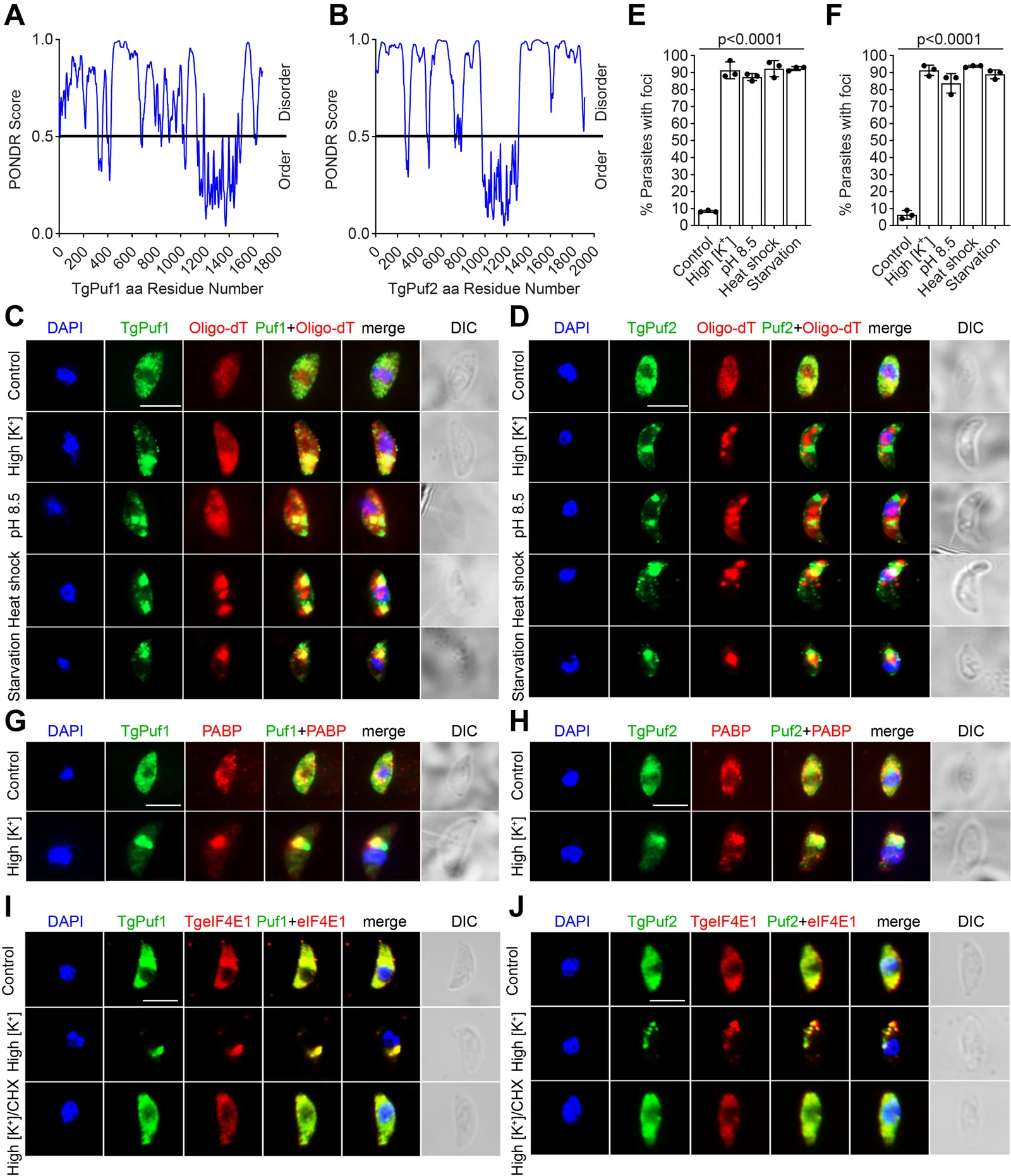
**Dynamic localization change of TgPuf proteins under stress conditions.***A* and *B*, the predicted disordered regions of TgPuf1 (*A*) and TgPuf2 (*B*) were identified using PONDR. *C*, *D*, Localization analysis of TgPuf1 (*C*) and TgPuf2 (*D*) proteins in RHΔ*ku80* parasites, determined by combined RNA-FISH, immunofluorescence under stress conditions (high K^+^ buffer, pH 8.5, heat shock, and starvation). DAPI (*blue*), TgPuf1/2 (*green*), and Cy5-labeled oligo dT primer (*red*). *E* and *F*, quantification foci containing parasites for TgPuf1 (*E*) or TgPuf2 (*F*) proteins, analyzed from fig. *C* and *D*. n = 3 replicates; mean ± S.D; one-way ANOVA; Dunnett’s comparison test; ns not significant; *p* values: ∗ <0.05; ∗∗∗ <0.001; ∗∗∗∗ <0.0001. *E*, *F*. *G* and *H*, colocalization of PABP and TgPuf1 (*G*) or TgPuf2 (*H*), analyzed in high K^+^ using immunofluorescence with α-TgPuf1, α-TgPuf2, and α-HsPABP antibodies. *I* and *J*, colocalization of TgeIF4E1 and TgPuf1 (*I*) or TgPuf2 (*J*), analyzed in *high* K^+^ ± 50 μg/ml cycloheximide (CHX) for 1 h, followed by immunofluorescence with α-TgPuf1, α- TgPuf2, and α-TgeIF4E1 antibodies and DNA by DAPI staining. Cycloheximide is used to control stress granule formation.

The typical Puf protein features a central RNA-binding domain, flanked by extended N-terminal and short C-terminal intrinsically disordered regions (IDRs). These IDRs play an important role in modulating RNA-binding activities and facilitating the assembly of higher-order ribonucleoprotein (RNP) complexes, such as membrane-less granules (54). Compared to model organisms, *Toxoplasma* Puf proteins are large in size (∼70 kDa *vs* ∼170 kDa) with a predicted structure available only for the Puf domain. Accordingly, TgPufs sequences were submitted to PONDR (55) to identify natural disordered regions in the protein. The analysis revealed that, except for the RNA-binding domain positioned centrally, the remaining N- and C-terminal regions of both the TgPuf1 (Figs. 5*A* and S6*A*) and TgPuf2 (Figs. 5*B* and S6*B*) proteins have high intrinsically disordered regions. *Toxoplasma* parasites encounter stressful conditions at various stages of their life cycle during transmission. Hence, to examine whether TgPuf proteins form RNP complexes, we exposed the parasites to various physiological stress conditions: starvation, heat shock, pH 8.5, and high [K^+^] (56, 57, 57). The combined RNA-FISH immunofluorescence analysis demonstrated that under various physiological stress conditions, both TgPuf1 (Fig. 5*C*) and TgPuf2 (Fig. 5*D*) proteins displayed a dynamic change in the localization from a diffuse to a punctate/foci pattern, primarily associated with RNA (Cy5-OligodT_45_). In total, ∼90% of the parasites exhibited foci of TgPuf proteins, regardless of the stress conditions present (Fig. 5, *E* and *F*). To further investigate the role of TgPuf proteins in the stress response, we used *bona fide* stress response proteins, PABP (Fig. S7) and eIF4E1 (15), and performed colocalization studies. Under normal conditions, both TgPufs and PABP/eIF4E1 proteins exhibited diffuse staining (Fig. 5, *G*–*J*). However, following treatment with high [K^+^], both proteins showed punctate staining with strong colocalization (Fig. 5, *G*–*J*). Additionally, we treated the parasites with cycloheximide (CHX), which is known to shift RNA granules from a punctate state to a diffuse cytoplasmic localization. We observed a dynamic change in the localization of both TgPuf proteins, from a punctate to a diffuse pattern, similar to that of TgeIF4E1 (Fig. 5, *I* and *J*). Collectively, these results demonstrate that TgPuf proteins form stress-induced cytoplasmic foci.

### TgPuf1, not TgPuf2, has a role in the survival of tachyzoites under stress conditions

**Figure 6:**
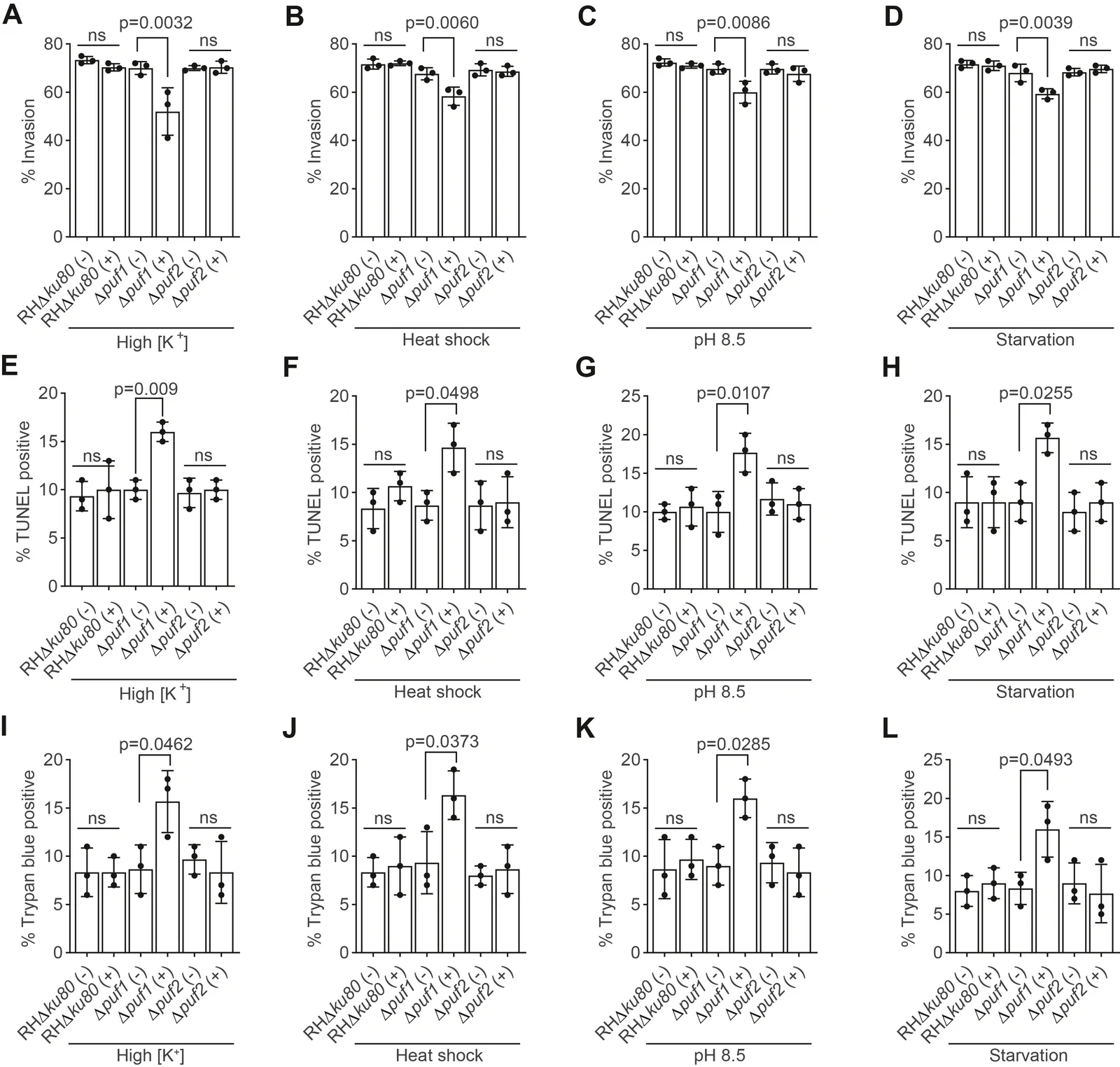
**Effect of stress on tachyzoite growth in parasites lacking TgPuf1 and TgPuf2.***A–D*, invasion abilities of the RHΔ*ku80*, RHΔ*puf1*, and RHΔ*puf2* strains were tested under different stress conditions: *high* K^+^ buffer (*A*), heat shock (*B*), pH 8.5 (*C*), and starvation (*D*) using an invasion assay. The graph shows the percentage of invasion for each parasite strain on the HFF monolayer. *E–H*, The death of the RHΔ*ku80*, RHΔ*puf1*, and RHΔ*puf2* strains was tested under various stress conditions: *high* K^+^ buffer (*E*), heat shock (*F*), pH 8.5 (*G*), and starvation (*H*) using the TUNEL assay. The graph shows the percentage of TUNEL-positive parasites, indicating the proportion of dead or dying parasites. *I–L*, parasite viability of the RHΔ*ku80*, RHΔ*puf1*, and RHΔ*puf2* strains were tested under different stress conditions: *high* K^+^ buffer (*I*), heat shock (*J*), pH 8.5 (*K*), and starvation (*L*) using trypan *blue* staining. The graph shows the percentage of trypan *blue-positive* parasites (dead parasites). n = 3; mean ± SD; one-way ANOVA; Tukey’s *post hoc* test; ns, not significant; *p* value: ∗ <0.05; ∗∗∗ <0.001; ∗∗∗∗ <0.0001.

TgPuf proteins are components of RNP complexes; however, understanding the effect of TgPuf protein deletion on parasite survival during stress conditions is essential. To examine this, RHΔ*puf1* and RHΔ*puf2* parasites were exposed to different physiological stress conditions, such as high [K^+^], heat shock, pH 8.5, and starvation, and their viability was evaluated. Initially, we assessed the invasion ability of these parasites. The results showed that RHΔ*puf1* parasites had a significantly reduced invasion ability, whereas the invasion efficiency of RHΔ*puf2* parasites was comparable to that of the parental RH strain (Fig. 6, *A–D*). Additionally, a significantly higher percentage of RHΔ*puf1* parasites (∼15%) showed DNA damage (TUNEL positive) than RHΔ*puf2* and parental parasites (∼10%) (Fig. 6, *E–H*). Finally, parasite death under these conditions was assessed using trypan blue staining. The death rate of parasites was twice as high in RHΔ*puf1* parasites (∼15%) compared to RHΔ*puf2* and parental parasites (∼7%) (Fig. 6, *I–L*). These results suggest that RHΔ*puf1* parasites are more prone to death under stress conditions, highlighting that TgPuf1 plays a role in parasite survival in the extracellular environment and during transmission.

### TgPuf proteins bind to the deadenylase enzyme, TgPop2

**Figure 7:**
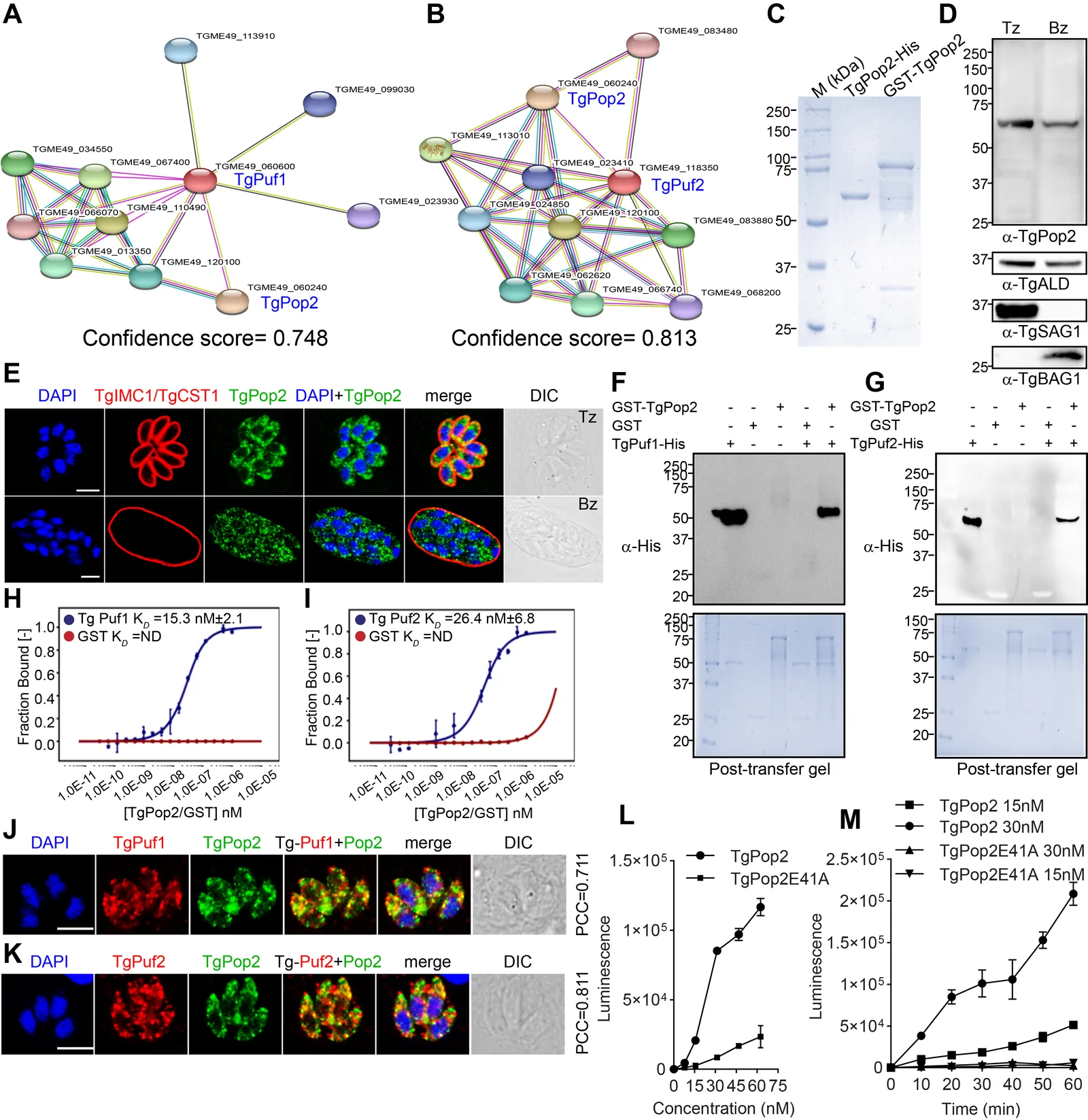
**TgPuf proteins interact with the deadenylase enzyme TgPop2.***A*, *B*, potential interacting proteins for TgPuf (1 and 2) were identified using the STRING database. Both proteins interact with putative TgPop2, a deadenylase protein with a high confidence score. *C*, purification profile of full-length recombinant TgPop2-His (∼64 kDa) and GST-TgPop2 (∼90 kDa) analysed on 10% SDS-PAGE. *D*, Expression levels of TgPop2 in tachyzoites and bradyzoites determined by immunoblotting using α-TgPop2 antibodies. Loading controls: TgALD - parasite proteins, TgSAG1 - tachyzoite stage marker, and TgBAG1 - bradyzoite stage marker. *E*, TgPop2 localization in tachyzoite and bradyzoite stages was analyzed by immunofluorescence using α-TgPop2 antibodies. The α-TgIMC1 antibody marks the tachyzoite inner membrane, while α-TgCST1 labels the bradyzoite cyst wall. Scale bar, 5 μm. *F* and *G*, GST pull-down assay using beads bound GST-TgPop2 or GST alone was incubated with TgPuf1-His and TgPuf2-His. The proteins bound to GST-TgPop2 and GST were analyzed by SDS-PAGE and immunoblotting using α-His antibodies. *H*, *I*, MST analysis of labelled His-TgPuf1 (*H*) and His-TgPuf2 (*I*) with GST-TgPop2, or GST alone. *J*, *K*, Immunofluorescence analysis of tachyzoites was performed using co-staining with α-myc/α-mouse IgG Fluor 594 and α-TgPop2/α-rabbit IgG Fluor 488. (Pearson’s Correlation Coefficient; TgPuf1 and TgPop2 = 0.711, TgPuf2 and TgPop2 = 0.811). Scale bar = 5 μm. *L*, deadenylase activity of recombinant TgPop2 and TgPop2E41A were assessed using increasing enzyme concentrations and 50 nM poly(A)20 RNA substrate (CAGACCUGUAUAUACACACU[A]_20_). AMP release was measured with the AMP-Glo Assay System. *M*, a time-course study was conducted with recombinant TgPop2 and TgPop2E41A proteins at concentrations of 15 nM and 30 nM, using a 50 nM poly(A)20 RNA substrate.

To explore the potential function of Puf proteins in the parasite, we performed STRING protein-protein interaction network analysis (58) for TgPuf1 and TgPuf2. The predicted TgPuf1 interactome (Fig. 7*A*) comprised 10 partners, while the TgPuf2 interactome (Fig. 7*B*) comprised 11 partners. Notably, both networks converged on a shared interactor annotated as a putative CCR4-family deadenylase (TGME49_260240, hereafter TgPop2) (Figs. 7, *A*, *B*, S8 and S9*A*), which was independently recovered as a high-confidence interaction partner of both TgPuf1 (confidence score of 0.748) and TgPuf2 (confidence score of 0.813). Because Pop2-family deadenylases function as the catalytic subunit of the CCR4-NOT deadenylase complex in other eukaryotes, directly linking RNA-binding proteins to mRNA decay, we selected TgPop2 for further functional characterization. To determine the role of putative TgPop2 and its interaction with TgPufs, we first bacterially expressed the full-length TgPop2 (His-tag, ∼64 kDa and GST-tag, ∼90 kDa) (Fig. 7*C*, Table S1) and generated anti-TgPop2 polyclonal antibodies by immunizing with TgPop2-His protein. The expression and localization studies using anti-TgPop2 antibodies demonstrated that TgPop2 is highly expressed (∼64 kDa) in both the asexual stages (Fig. 7*D*) and is localized in the cytoplasm of the tachyzoite and bradyzoite stages (Fig. 7*E*). To examine the interaction between TgPuf and TgPop2, a GST pull-down assay was performed. The results clearly demonstrated that GST-TgPop2 can efficiently pull down TgPuf1/2-His, confirming their interaction (Fig. 7, *F* and *G*). Further, this interaction was quantitatively assessed using MST, which showed that the TgPuf1 (K_*D*_ = 15 nM) and TgPuf2 (K_*D*_ = 26 nM) proteins interact with TgPop2 with high affinity (Fig. 7, *H* and *I*). To further support these results, we performed IFA on tachyzoites, revealing strong colocalization of TgPop2 with TgPuf1 (Pearson’s correlation coefficient = 0.711) (Fig. 7*J*) and TgPuf2 (Pearson’s correlation coefficient = 0.811) within the cytoplasm of the parasites (Fig. 7*K*).

Further, we examined the deadenylase activity of TgPop2 using a detailed biochemical characterization. Pop2 proteins belong to the conserved Caf1 family of DEDD type 3′-5′ exonucleases, which are defined by four invariant acidic residues (D-E-D-D) that coordinate the catalytic activity of the Pop2 enzyme (59, 60, 60). Structural and biochemical studies of Caf1/Pop2 homologs have shown that the conserved glutamate (E) (59) in this motif directly participates in magnesium ion coordination and is essential for RNA hydrolysis. The glutamate is also conserved in TgPop2 (Fig. S9*B*), and therefore, to test whether TgPop2 uses the same catalytic mechanism, we generated a point mutant in which the conserved glutamate (E) at position 41 was replaced with alanine (A) (TgPop2E41A) (Fig. S10, *A*–*C*).

The recombinant TgPop2, at concentrations ranging from 0 to 60 nM, demonstrated the ability to catalyze the release of AMP from a poly(A)20 RNA substrate containing the PRE sequence (Fig. 7*L*); however, TgPop2E41A showed significantly reduced activity at these concentrations. The deadenylation activity of TgPop2 reached saturation at a concentration of 200 nM. (Fig. S11). The release of AMP was also confirmed using the conventional method of utilizing radiolabelled RNA substrate (Fig. S12). Due to significantly high luminescence readings at protein concentrations >30 nM (Fig. 7*L*), we decided to use concentrations of 15 nM and 30 nM of TgPop2 for the subsequent time kinetics experiments. Both concentrations showed effective AMP release within 30 min of incubation in wild-type TgPop2, unlike TgPop2E41A (Fig. 7*M*).

### TgPuf proteins stimulate deadenylation activity of TgPop2 and promote the degradation of PRE-containing transcripts

**Figure 8:**
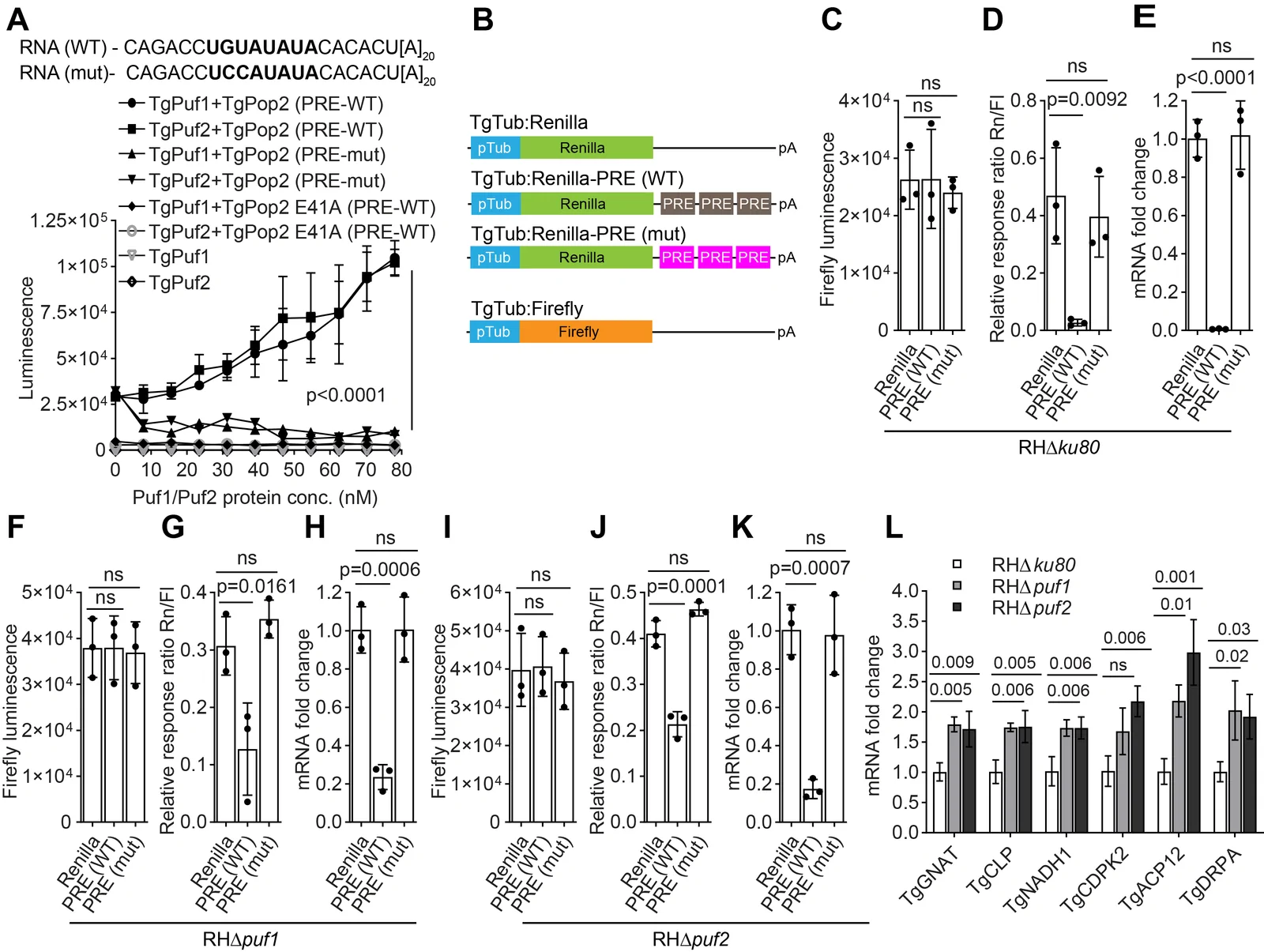
**The impact of the TgPuf-TgPop2 interaction of PRE-transcripts.***A*, the release of AMP from a wild-type (WT) or mutant PRE with a poly(A)20 substrate was assessed using recombinant TgPuf1 or TgPuf2 at varying concentrations (0–80 nM) while keeping TgPop2 constant at 15 nM. Control reactions used TgPuf1 or TgPuf2 alone. *B*, schematic illustration shows the generation of luciferase constructs. 1. Renilla, 2. Renilla with 3xPRE (WT), 3. Renilla with 3xPRE (mut), and 4. Firefly. All constructs were generated by cloning the respective sequences into pTUB1:YFP-mAID-3HA plasmid. *C*, *F*, *I*, FLuc levels (RLU) in the RHΔ*ku80* (*C*), RHΔ*puf1* (*F*), or RHΔ*puf2* (*I*) parasites cotransfected with FLuc and RLuc or RLuc PRE (WT), or RLuc PRE (mut). *D*, *G*, *J*, relative response ratio of RLuc/FLuc (Rn/Fl) in the RHΔ*ku80* (*D*), RHΔ*puf1* (*G*), or RHΔ*puf2* (*J*) parasites cotransfected with FLuc and RLuc or RLuc PRE (WT), or RLuc PRE (mut). *E*, *H*, *K*, mRNA levels of RLuc, RLuc PRE (WT), RLuc PRE (mut) in the RHΔ*ku80* (*E*), RHΔ*puf1* (*H*), or RHΔ*puf2* (*K*) parasites, determined by RT-qPCR. *L*, mRNA levels of TgGNAT, TgCLP, TgNADH1, TgCDPK2, TgACP12, and TgDRPA in the RHΔ*ku80*, RHΔ*puf1*, or RHΔ*puf2* parasites, determined by RT-qPCR. n = 3 replicates; mean ± SD; two-way ANOVA with Tukey’s *post hoc* test; ns not significant; *p* values: ∗ <0.05; ∗∗∗ <0.001; ∗∗∗∗ <0.0001.

To assess the biological significance of the interaction between TgPufs and TgPop2, a deadenylation assay was performed for TgPop2/TgPop2E41A with or without TgPufs and synthetic poly(A)20 RNA containing either the wild-type or mutant PRE sequence. The results showed a significant increase in the deadenylase activity of TgPop2 as the concentration of TgPufs (0–80 nM) increased in reactions containing RNA with the WT PRE (Fig. 8*A*). The deadenylase protein TgPop2, which removes adenine from the poly(A) tail of RNA, was confirmed using a conventional method with radiolabeled RNA substrate (Fig. S13). Interestingly, the basal deadenylase activity of TgPop2 was decreased in reactions with RNA containing the mutant (mut) PRE (Fig. 8*A*), suggesting that free TgPufs bind to TgPop2, and thereby inhibiting its activity. The degradation of RNA containing WT PRE, as observed in the deadenylase assay, was further examined using a reporter assay. Reporter plasmids were constructed by incorporating the Renilla luciferase gene (RLuc), or the firefly luciferase gene (FLuc), or RLuc followed by 3x-PREs (wild type or mutant), under the control of the TgTub promoter (Fig. 8*B*, Table S1). After cotransfection of RLuc and FLuc, or RLuc+3x-PREs (WT) and FLuc, or RLuc+3x-PREs (mut) and FLuc, in RHΔ*ku80*, RHΔ*puf1*, or RHΔ*puf2* parasites, luciferase activity was measured (Fig. 8, *C–K*). In RHΔ*ku80*, RHΔ*puf1*, and RHΔ*puf2* parasites, the FLuc activity remained consistent across all three FLuc constructs (Fig. 8, *C*, *F* and *I*), as expected. In the same experimental conditions; however, the Rn/Fl ratio was significantly reduced in RHΔ*ku80* parasites (∼90%, Fig. 8*D*) compared to RHΔ*puf1* (∼60%, Fig. 8*G*), and RHΔ*puf2* (∼60%, Fig. 8*J*) parasites when cotransfected with RLuc+3x-PREs (WT) and FLuc. This Rn/Fl ratio also aligns with the relative mRNA levels of RLuc (Fig. 8, *E*, *H* and *K*, Table S1). Notably, the Rn/Fl ratio for RLuc+3x-PREs (mut) and FLuc (Fig. 8, *D*, *G* and *J*) and the relative mRNA levels of RLuc (Fig. 8, *E*, *H* and *K*) remained unchanged irrespective of parasite strain. To validate the synthetic reporter results, the mRNA levels of several candidate PRE-containing genes, including TgGNAT, which harbours up to 13 PRE sites, were assessed. Similar to reporter genes, the mRNA levels of six (TgGNAT, TgCLP, TgNADH1, TgCDPK2, TgACP12, and TgDRPA) randomly selected PRE-containing genes (Table S1 and S2) tested were significantly higher in RHΔ*puf1* or RHΔ*puf2* parasites than in RHΔ*ku80* (Fig. 8*L*). Together, these findings suggest that TgPufs may facilitate the degradation of PRE-containing transcripts through TgPop2 in the parasites.

### TgPuf1 and TgPuf2 double mutant is potentially lethal to the parasite

**Figure 9:**
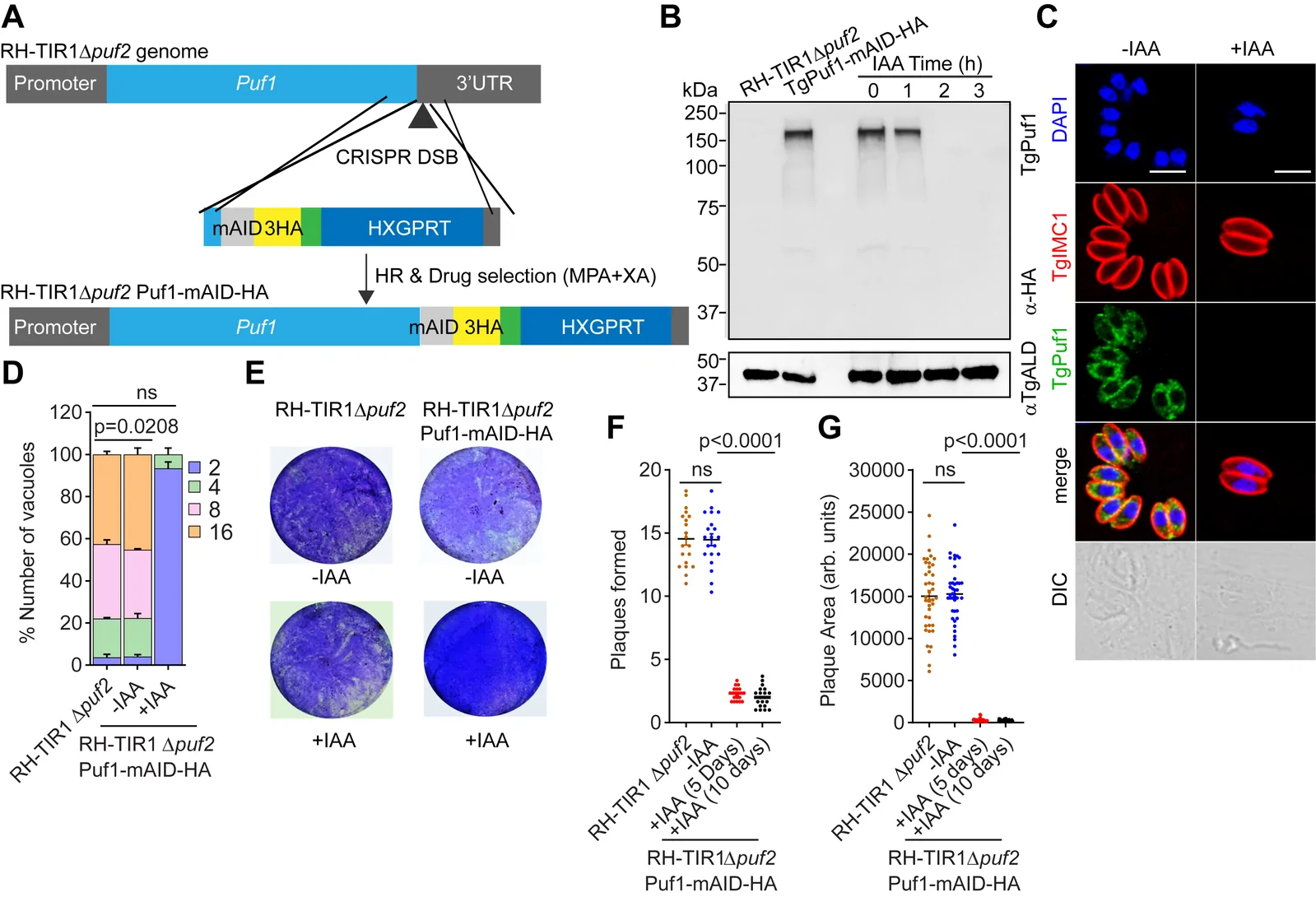
**Effect of double mutant (TgPuf1 and TgPuf2) on parasite replication.***A*, strategy for mAID-HA tagging of TgPuf1 protein in RH-TIR1 Δ*puf2* parental line (RH-TIR1Δ*puf2* Puf1-mAID-HA). *B*, Western blot analysis using anti-HA antibodies determined expression of TgPuf1-mAID-3HA in RH-TIR1 Δ*puf2*-transfected parasites, with or without IAA, at the indicated time points. *C*, immunofluorescence analysis of TgPuf1-mAID-HA grown with IAA or vehicle for 2 h (n = 3). Scale bar 5 μm. *D*, Parasite replication. The graph shows the percentage of vacuoles containing the number of parasites of RH-TIR1, RH-TIR1 Δ*puf2* and RH-TIR1Δ*puf2* Puf1-mAID-HA parasites treated for 18 h with IAA or vehicle (n = 3) determined by IFA. *E*, plaque assay. Crystal violet-stained images of plaques formed by RH-TIR1, RH-TIR1 Δ*puf2* and RH-TIR1Δ*puf2* Puf1-mAID-HA parasites on HFF monolayer treated with IAA or vehicle (n = 3). *F*, *G*, quantification of plaque numbers (*F*) and plaque areas (*G*). *H*. IFA showing expression of TgPuf1 and TgCST1 in RH-TIR1Δ*puf2* Puf1-mAID-HA parasites (−IAA/+IAA on day 0, 2, 4) (n = 3). *D-G*; n = 3 replicates; mean ± S.D; one-way ANOVA; Dunnett’s comparison test; ns not significant; *p* values: ∗ < 0.05; ∗∗∗ < 0.001; ∗∗∗∗ < 0.0001.

Both TgPuf’s similarities in RNA binding and localization and its activation of TgPop2 suggest a possible compensatory role. To test this, we generated a TgPuf1-mAID conditional knockdown (Fig. 9*A*) in the RH-TIR1Δ*puf2* parasites (RH-TIR1Δ*puf2* Puf1-mAID-HA) and tested the essentiality of both proteins by conditionally depleting TgPuf1 with IAA treatment. We observed complete loss of the TgPuf1-mAID-HA protein after 2 h of IAA treatment by western blotting (Fig. 9*B*) and IFA (Fig. 9*C*). The loss of both TgPuf proteins, leading to arrest of parasite replication with vacuoles showing significantly high number two parasites compared to parental strain (Fig. 9*D*). Additionally, the double mutant showed significant reduction in the parasite viability, as very few small plaques were observed on day 5 with no further increase either the number or size till day 10 (Fig. 9, *E–G*). These results suggest that the double mutant could be lethal to the parasite, and both Puf proteins may have overlapping functions.

We then investigated whether the loss of both TgPuf proteins induces bradyzoite formation, in addition to the severe growth defect observed in the parasite. To examine spontaneous bradyzoite formation following the depletion of TgPuf1, we cultured RH-TIR1Δ*puf2* Puf1-mAID-HA parasites in normal medium containing IAA and monitored them for up to 4 days using immunofluorescence with the TgCST1 antibody (Fig. 9*H*). The RH strain does not naturally differentiate into bradyzoites under standard conditions, but can do so under stress, like alkaline pH and CO_2_ depletion. Hence, RH-TIR1Δ*puf2* Puf1-mAID-HA parasites were cultured in alkaline medium for 4 days and compared the resulting parasites with those cultured in the presence of IAA (Fig. 9*H*). As expected, TgCST1 staining was observed in untreated parasites on days 2 and 4. However, no TgCST1 staining was detected in IAA-treated parasites on either day (Fig. 9*H*). This suggests that the loss of both TgPuf proteins does not induce bradyzoite formation, at least *in vitro*, for the period tested.

## Discussion

Translational regulation of mRNA is an immediate and precise mechanism for controlling gene expression, driven by the interaction of RNA-binding proteins with specific sequence elements in the UTRs of mRNA, thereby influencing mRNA stability, localization, and translational efficiency. In this study, we identified the Puf family of RBPs in *Toxoplasma* and functionally characterized two Puf proteins, TgPuf1 and TgPuf2, during the asexual stages (tachyzoite and bradyzoite). Deletion of TgPuf1 marginally reduces parasite replication and viability under stress conditions, whereas TgPuf2 knockout has no discernible effect on parasite replication or viability. Both TgPuf proteins bind the conserved PRE-containing RNA sequence, are components of cytoplasmic ribonucleoprotein complexes, and interact with the deadenylase enzyme TgPop2. This interaction modulates TgPop2's deadenylase activity, promoting RNA degradation. Our attempts to generate double-mutant parasites for Puf1 and Puf2 were unsuccessful, suggesting that the absence of these proteins may be essential for parasite fitness. Moreover, the modest phenotypes observed in the single mutants might underestimate the full regulatory scope of PUF-mediated regulation concerning parasite fitness and stress adaptation. Since both TgPuf proteins are localized in the cytoplasm and bind to the same RNA sequence with similar affinities, it is reasonable to speculate that they might have overlapping roles. This is reminiscent of the roles of Pum1 and Pum2 in mice, where a double knockout of these proteins is lethal (65).

Most eukaryotic lineages encode at least one of three Puf protein types (Puf, Nop9, and PUM3), each with distinct biological roles and structural features (34, 35, 36, 37, 38). We found that, with a few exceptions, apicomplexan parasites, including *Toxoplasma*, also harbor all three Puf protein types. Like other Puf proteins, both classical TgPuf1 and TgPuf2 are characterized by 8 Puf repeats and are predicted to adopt a crescent-shaped structure, whereas Nop9 and PUM3 consist of 11 Puf repeats arranged in C-shaped and L-shaped folds, respectively. Despite their critical role in regulating transcript levels, neither of the TgPuf proteins is essential for the asexual stages of parasites. TgPuf1 appears to be important under stress conditions, as evidenced by the reduced fitness of RHΔ*puf1* parasites, while TgPuf2 is not important under the same conditions. Given the diverse roles of classical Puf proteins throughout the developmental stages of eukaryotes, it is tempting to speculate that both TgPuf proteins may be important during the sexual developmental stages that occur in the feline definitive host. The existence of 32 genes containing the PRE motif, which are expressed during these sexual stages, substantiates this hypothesis. Furthermore, the functionality of classical Puf proteins has primarily been demonstrated during the sexual stages of *Plasmodium* species (44, 45, 46, 47, 48, 49), further reinforcing this assumption.

Puf proteins are essential for invertebrates (*Drosophila* and *Caenorhabditis elegans*), as they regulate the development of early embryos and germline stem cells (62, 63, 63). In mammals, specifically mice, there exist two Puf proteins: Pum1 and Pum2; single knockout of either gene results in viable, fertile mice (64, 65, 66), whereas a double knockout leads to embryonic lethality (61). Yeast *S*. *cerevisiae* has six Puf proteins (34) that are non-essential for growth but are important under stress (61, 61, 67, 68, 69, 70, 71). A protozoan, *Trypanosoma* *brucei*, contains 11 Puf proteins (35), and all except Puf4 and Puf5 are essential for growth, differentiation, or rRNA processing. Apicomplexan, *P*. *falciparum*, contains three Puf proteins, Puf1 (46), Puf2 (47, 48, 49), and Puf3 (50), which are essential for either female gametogenesis, sporozoite development, or ribosomal biogenesis, respectively. In contrast, in *P*. *berghei*, Puf1 is dispensable, while Puf2 prevents premature sporozoite transformation and is critical for transitioning between the insect and the mammalian hosts (45). In the cattle parasite *Neospora caninum*, Puf1 is not essential for stage differentiation; however, its deletion impairs parasite replication *in vitro* and virulence in mice (72). Overall, Puf proteins can be essential or non-essential in different organisms, often exhibiting redundancy due to overlapping functions. For example, in mice, Pum1 and Pum2 exhibit redundancy (64, 65, 66), and in *C*. *elegans*, FBF-1 and FBF-2 have overlapping roles (73). The observation that the double mutant of Pufs is potentially lethal and that a Renilla reporter containing PRE was expressed at slightly higher levels in RHΔ*puf1* or RHΔ*puf2* parasites compared to parental RH parasites, also suggests overlapping functions of Puf proteins in *T*. *gondii*. However, the lack of change in TgPuf1 expression in RHΔ*puf2* or *vice versa* compared to parental RH parasites complicates this conclusion.

Puf proteins are rich in IDRs, which play a crucial role in regulating RNA-binding affinity and protein-protein interactions necessary for assembling large RNP complexes, such as membrane-less granules (54). During cellular stress, these granules assemble to sequester RNA and proteins, regulating mRNA metabolism by preventing translation or initiating degradation (74, 75, 75). In eukaryotes, non-translating mRNAs are typically packaged into processing bodies (P-bodies, PBs) and stress granules (SGs) (54, 74, 75, 74, 75). PBs contain proteins involved in mRNA decay, while SGs form in response to stress conditions (54, 74, 75, 74, 75). Despite their differences, both types of mRNP granules share various RBPs, including Puf proteins (74, 75, 75). Our knowledge of these RNPs, including their composition and interactions, is very limited in apicomplexan parasites. The formation of mRNP granules, particularly SGs, has been observed in *Toxoplasma* both before parasite egress and in extracellular parasites (76, 77, 77). Intriguingly, typical SG markers, such as PABP and DEAD-box RNA helicases, were not detected in the proteomes of RNP granules from extracellular parasites (78), suggesting challenges in isolating SGs or limitations of the methodology. Through STRING network analysis of TgPuf proteins, we identified proteins associated with both PB and SG. Similarly, in *P*. *falciparum*, the interactome of the DEAD-box helicase PfDOZI, a protein involved in PB formation, showed enrichment for proteins found in both PB and SG (79), suggesting their coexistence and may serve as a platform either to inhibit mRNA translation or to promote transcript degradation *via* TgPuf-Pop2 interactions (for PRE-containing transcripts). Compared to model organisms, *Toxoplasma* Puf proteins (Puf1 and Puf2) are considerably larger, with extensive disordered regions (except the PUM-HD) contributing up to 75% of the total protein. Therefore, it is imperative to determine the extent to which the IDRs contribute to the dynamic localization of TgPuf proteins, the formation of these RNP complexes, and the subsequent fate of mRNA within such structures.

In model organisms, Ccr4 and Pop2, both possessing ribonuclease activity, form the catalytic core of the Ccr4-NOT deadenylase complex. Through a homology search, we identified the Ccr4 gene (TGME49_259560) in *Toxoplasma*. Given its CRISPR score of −0.46, Ccr4 (80) does not appear to be essential, suggesting that Pop2 (CRISPR score −2.4) (80) may serve as the primary deadenylase in *Toxoplasma*; however, this hypothesis needs to be tested experimentally. The possibility of an essential role for TgPop2 also aligns with yeast, where *pop2* mutants show impaired deadenylation and repression of target mRNAs, whereas *ccr4* mutants exhibit only a modest effect on repression (81). Additionally, the effect of TgPuf deletion on the poly(A) tail of the target mRNAs should be assessed alongside Pop2 deletion to determine whether it affects the basal rate of poly(A) tails removal.

RNA modifications such as pseudouridine (Ψ) and *N*^6^-methyladenosine (m^6^A) weaken interactions between the human PUM2 protein and RNA (82). Ψ and m6A are the most prevalent mRNA modifications in eukaryotes, with human cells containing 100 to 1000 Ψ sites (83) and about 12,000 m^6^A sites (84). To date, Ψ (85) and m6A (86, 87, 87) are the only two mRNA modifications reported in *Toxoplasma*. It will be interesting to determine whether these Ψ and m6A modifications affect RNA binding of TgPuf proteins and whether this effect is similar to the weaker protein binding observed for the human PUM2 protein *in vitro*. Given the high abundance of Ψ and m6A and their partial overlap with the PUF-binding site, it is tempting to speculate that multiple mRNA targets of TgPufs during the asexual stages are likely modified and may be protected from degradation by TgPop2 due to reduced binding of TgPuf proteins to their targets.

In summary, these findings demonstrate that both TgPuf proteins regulate transcript levels by binding to the same RNA sequence with similar affinities. While the absence of one protein is not detrimental, the double mutant is essential, suggesting that these proteins have overlapping functions in *Toxoplasma*.

## Experimental procedures

### Ethics statement

The ethics committee at the National Institute of Animal Biotechnology has approved the use of laboratory research protocols (IBSC/Feb2023/NIAB/AD001) and animal research (IAEC/NIAB/2024/09/ASD).

### Parasite culture

*T. gondii* strains RHΔ*ku80*Δ*hxgprt* (88), ME49, RH/ME49-Δ*puf1*, RH/ME49-Δ*puf2*, RH-TIR1 (89), RH-TIR1Δ*puf2*, and RH-TIR1Δ*puf2* Puf1-mAID-HA were maintained in primary human foreskin fibroblasts (HFF, ATCC) cells in Dulbecco's modified Eagle's medium (DMEM) supplemented with 10% FBS, 10 μg/ml gentamicin, and 2 mM L-glutamine at 37 °C and 5% CO2 in a humidified incubator. To obtain purified parasites for experiments, parasite-infected HFF cells were scraped and passed through a 27-gauge needle. The released parasites were then filtered through a 3.0 μm filter to separate them from host cell debris.

### Gene cloning, expression, and protein purification

The synthetic DNAs encoding TgPuf1 (RBD: 3079–4536 bp), TgPuf2 (RBD: 2641–4146 bp), and full-length TgPop2 (1854 bp) genes were synthesized by Life Technologies. Initially, all three genes were cloned into the pMS vector (Life Technologies) between the *Nde*I and *Xho*I, sites and were subsequently subcloned into either pET-21a or pGEX-6P-2 vectors. The recombinant proteins were expressed in *E*. *coli* BL21 Rosetta with a C-terminal 6xHis-tag (TgPuf1 ∼53 kDa, TgPuf2 ∼55 kDa, and TgPop2 ∼64 kDa) or N-terminal GST-tag (TgPop2 ∼90 kDa) and purified using nickel nitrilotriacetic acid-agarose or GSH-agarose resins, as previously described (90, 91, 91). Briefly, *E*. *coli* transformed with TgPuf1, TgPuf2, or TgPop2 was grown in 10 ml of Luria-Bertani (LB) medium supplemented with 50 μg/ml ampicillin and 34 μg/ml chloramphenicol overnight at 37 °C. The overnight culture was transferred to 1000 ml of LB with the same antibiotics and incubated at 37 °C with shaking. Once the OD_600_ reached 0.4, IPTG was added to a final concentration of 1 mM, and incubation continued at 25 °C for 16 h. Following incubation, the cells were harvested by centrifugation, and the pellets were resuspended in 40 ml of lysis buffer [50 mM NaH_2_PO_4_, 300 mM NaCl, 100 μg/ml lysozyme, 10 mM imidazole (added only for His-tag protein purification), 0.1% Triton X-100, and 0.3 mM PMSF, pH 8.0]. After centrifugation at 15,000×*g*, the supernatant was collected, and the protein was purified using Ni2+-NTA agarose (Qiagen) and eluted using 300 mM imidazole. The GST-tagged protein was purified using GSH-agarose resin and eluted using 10 mM reduced glutathione. The quality of the eluted protein was analyzed using SDS-PAGE. The purified recombinant proteins were dialyzed in 1X PBS and stored at −80 °C.

TgPop2E41A was generated by site-directed mutagenesis using a gene-specific set of primers (Table S1) and the Stratagene QuikChange (#210515) site-directed mutagenesis protocol. The resulting mutant construct was sequence-verified, and the TgPop2E41A recombinant protein was purified using the same protocol as the wild-type TgPop2 protein.

### Polyclonal antibody raising

Polyclonal antibodies against recombinant TgPuf1 (3079–4536 bp/1027–1512 aa), TgPuf2 (2641–4146 bp/881–1382 aa), and TgPop2 (1854 bp/618 aa) were generated through an initial injection of 25 μg of purified recombinant protein in Freund's complete adjuvant (Sigma #F5881) for mice or 50 μg for rabbits. This was followed by four booster injections of 10 μg (for mice) or 15 μg (for rabbits) each, administered in Freund's incomplete adjuvant (Sigma #F5506) at 2-week intervals. Serum was collected 60 days after the initial immunization. Polyclonal antibodies for Tg-SAG1 (92), IMC1 (93), Aldolase (ALD) (94), BAG1 (95), CST1 (92), and eIF4E1 (95) were used from our previous studies.

### Generation of TgPuf1-myc and TgPuf2-myc–tagged parasites

A 3xmyc epitope was inserted at the C-terminus of the TgPuf1 and TgPuf2 proteins using a genetic knock-in approach (88). To create C-terminally 3xmyc-tagged strains of TgPuf1 and TgPuf2, 1.5 kb and 1.9 kb DNA fragments upstream of the stop codons of *puf1* and *puf2*, respectively, were amplified and cloned into a pLIC-3xmyc-DHFR plasmid. The resulting plasmid was linearized using the restriction enzymes *Blp*I (for Puf1) and *BstB*I (for Puf2) and introduced into 10^7^ RHΔ*ku80* or ME49 parasites by electroporation using the Gene Pulser Xcell Total System (Biorad, #1652660). The 3xmyc tag was inserted at the end of the TgPuf1 and TgPuf2 genes by single-crossover recombination (88). The resulting transfectants were selected with 3 μM pyrimethamine and subsequently cloned. The final strains were named TgPuf1-myc and TgPuf2-myc.

### *In vitro* bradyzoite induction

HFF monolayers on a coverslip were infected with *T*. *gondii* tachyzoites (ME49) for 12 h at 37 °C. Differentiation to the bradyzoite stage was induced by replacing the DMEM with bradyzoite induction medium (RPMI pH 8.5, 1% FBS, and 10 μg/ml gentamicin) and incubating at 37 °C without CO_2_ for 3 days. Anti-CST1 antibodies were used to stain CST1 protein present in the cyst wall of bradyzoites.

### Immunoblotting

Filter-purified 10^5^ parasites were suspended in SDS-PAGE sample buffer, boiled for 10 min, and run on a single lane of a 10% polyacrylamide gel. The gel was then transferred to a 0.2 μm PVDF membrane using a Trans-Blot System (Bio-Rad) for 2:30 h at 100 V. The membrane was blocked in 5% non-fat milk in PBS for 60 min and subsequently probed overnight at 4 °C with primary antibodies [α-myc (#M4439, Merck Millipore), TgPuf1, TgPuf2, TgPop2, TgSAG1, TgBAG1, TgALD, or HsPABP (#PA5-29883, Invitrogen) −1:1000 in PBS; α-myc-1:5000 in PBS]. The membrane was washed 3× with PBS plus Tween-20 detergent (PBST; 0.1% Tween-20) and probed with HRP-conjugated α-rabbit IgG (#Sc-2357, Santa Cruz) or α-mouse IgG (#Sc-2005, Santa Cruz) antibodies - 1:5000 in PBS. The blot was developed using the Super Signal West Pico PLUS (ThermoScientific) and visualized on a ChemiDoc Imager (Bio-Rad).

### Immunofluorescence (IF) staining

HFF monolayers were grown on coverslips and infected with tachyzoites of RHΔ*ku80*, RHΔ*puf1*, or RHΔ*puf2*. Cells were fixed and permeabilized in PBS containing 4% paraformaldehyde and 0.05% Triton X-100 after 18 h of tachyzoite infection or 5 days of bradyzoite infection. Next, cells were blocked with 5% BSA and incubated sequentially with primary antibodies 1:100/PBS (α-myc, α-TgPuf1, α-TgPuf2, α-TgIMC1, α-TgCST1, α-TgPop2, α-HsPABP, or eIF4E1). After five washes with PBS, cells were incubated with secondary antibodies 1:1000/PBS [Alexa Fluor-488 (Mouse #A11001, Invitrogen; Rabbit #A11008, Invitrogen) or 594-conjugated α-mouse IgG (#A11005, Invitrogen) or α-rabbit IgG (#A11012, Invitrogen) or Alexa Fluor 647 (Mouse #A21235, Invitrogen) −1:1000 dilution]. Coverslips were mounted with Vectashield medium (Vector Labs) containing 4′, 6-diamidino-2-phenylindole dihydrochloride (DAPI) on a glass slide. Images were captured with a Leica Confocal microscope with a 63× objective and processed with LAS X software. For the cycloheximide (CHX) experiment, parasites were treated with high-K^+^ ± 50 μg/ml CHX (76) for 1 h immediately prior to fixation to assess stress granule formation.

For bradyzoite immunofluorescence, HFF monolayers grown on coverslips were infected with 10^3^ tachyzoites of ME49, ME49Δ*puf1*, or ME49Δ*puf2* and incubated for 12 h. Subsequently, tachyzoite to bradyzoite stage conversion was initiated by replacing the standard DMEM with bradyzoite induction medium, as detailed in the *in vitro* bradyzoite induction method section. The coverslips were then processed for immunofluorescence using antibodies against TgCST1 and either TgPuf1 or TgPuf2. TgCST1, a protein that marks the cyst wall, was used to quantify the number of bradyzoite cysts and measure their sizes. Cyst numbers were determined by counting cysts in 50 random fields for each experiment (n = 3). The size of the cysts was determined by measuring their area in square microns using Image J.

### Identification of PRE-containing genes

Annotated 3′UTRs and 5′UTRs were extracted from the *T*. *gondii* ME49 genome version 68 using the GFF3 feature types three_prime_UTR and five_prime_UTR. Corresponding nucleotide sequences were retrieved from the ME49 genome FASTA using R (v4.2.3) https://cran.r-project.org/bin/windows/base/old/4.2.3/running within RStudio (v0.17.1) http://www.rstudio.com/, using Bioconductor (v3.16) packages (96) GenomicRanges (v1.50.2) and Biostrings (v2.66.0) with supporting packages IRanges (v2.32.0), S4Vectors (v0.36.2), GenomeInfoDb (v1.34.9), and XVector (v0.38.0). UTR coordinates were imported as GRanges objects, and strand-aware sequence extraction was performed using Biostrings to ensure correct transcript orientation. UTRs consisting entirely of ambiguous bases (*e.g.*, “NNNN…”) were excluded from downstream analyses. The canonical Pumilio recognition element (PRE) was defined as UGUANAUA in RNA, corresponding to TGTANATA in DNA. PRE occurrences were counted using vcountPattern (Biostrings) with degenerate matching allowed. To estimate the expected random frequency of PRE matches, each UTR sequence was shuffled 100 times using a custom nucleotide-permutation function that preserves mono-nucleotide composition. PRE counts were recalculated for all shuffled sequences to determine the background motif probability p. For each UTR, a binomial probability was computed for observing k or more PRE matches given: 1. background motif probability p, and 2. number of motif windows (UTR length − motif length + 1). *p*-values were corrected using the Benjamini–Hochberg false discovery rate (FDR). UTRs with FDR ≤ 0.05 and at least 1 PRE occurrence were classified as high-confidence PRE-positive. Exact motif sequences and their positions were retrieved using matchPattern.

### Microscale thermophoresis (MST) assay

MST experiments were conducted with a Monolith NT.115 (NanoTemper) (97). The 100 nM of each of 5′-cyanine-labeled DmNRE; TgPRE (WT), or TgPRE (mut) (Table S1) was titrated against 0.01 nM- 400 nM concentrations of TgPuf1 or TgPuf2 protein. The reaction mixtures were incubated at 25 °C for 15 min in a binding buffer (50 mM Tris pH 7.4, 150 mM NaCl, and 5 mM MgCl_2_) and then loaded into glass capillaries (NanoTemper). The fluorescence intensity for each reaction was measured at 23 °C using 15% LED power and 40% MST power. The fluorescence values were analyzed using Affinity Analysis software version 2.3 (NanoTemper) to determine the binding affinity (dissociation constant: K_*D*_) between TgPuf1 or TgPuf2 and different RNA variants. The K_*D*_ is estimated by fitting the equation.

$$f\left(C\right)=U+\frac{\left(B-U\right)\times \left(C+T+K_{d}-\sqrt{{\left(C+T+K_{d}\right)}^{2}-4CT}\right)}{2T}$$

where C is concentration, U is unbound, B is bound, and T is target concentration (98).

For protein–protein interactions, His-tagged TgPuf1 and TgPuf2 proteins were labeled with RED-MALEIMIDE (second-generation dye), following the protocol provided by NanoTemper. A concentration of 25 nM of either TgPuf1 or TgPuf2 protein was titrated against GST-TgPop2 or GST protein at concentrations ranging from 0.030 nM to 1000 nM in a binding buffer (50 mM Tris pH 7.4, 150 mM NaCl, and 5 mM MgCl_2_). After 15 min of incubation at 25 °C, the reactions were loaded into glass capillaries, and fluorescence intensity measurements and analysis were performed as described above.

### GST pull-down assay

For GST pull-down experiments (89), GST-sepharose beads bound to 2 μg each of GST-TgPop2 or GST proteins were incubated with 1 μg each of TgPuf1-RBD or TgPuf2-RBD protein in a binding buffer containing 20 mM Tris, pH 8.0, 50 mM NaCl, 10% glycerol, and 0.3 mM PMSF, at 4 °C for 1 h. The protein-bound GST beads were washed thrice, followed by boiling in SDS sample buffer and analysis by SDS-PAGE and Western blot using anti-His antibodies.

### TgPop2 deadenylase activity

Deadenylase activity of TgPop2 and TgPop2E41A were assayed by quantifying the release of AMP (99, 100, 100). Standard reaction mixtures (20 μl) containing 40 mM Tris (pH 8.0), 100 mM NaCl, 2 mM MgCl2, 5% (v/v) glycerol, 2 mM DTT, 50 nM RNA substrate (synthetic poly(A)20 RNA substrate containing the PRE sequence purchased from GenScript), 15 nM/30 nM TgPop2, and TgPuf1/TgPuf2 as specified (0–80 nM) were incubated at 30 °C for 30 min. AMP release was detected using the AMP-Glo Assay (Promega, #V5011). Briefly, TgPop2 reactions were terminated by adding 20 μl of AMP-Glo Reagent I and incubating at 25 °C for 1 h. Then, 40 μl of AMP detection solution was added, followed by another hour of incubation at 25 °C. The luminescence readings were then measured using an EnSpire Multimode Plate Reader.

### Generation of TgPuf1 and TgPuf2 knockout parasites

TgPuf1 and TgPuf2 knockout parasites were generated using the CRISPR-Cas9 system by targeting guide RNAs to the gene upstream (89, 101, 101). The guide RNA sequence for the uprt gene in the plasmid pSAG1::CAS9-U6::sgUPRT (Addgene, #54467) was replaced with TgPuf1 guide RNA(pSAG1::CAS9-U6::sgPuf1) and TgPuf2 guide RNA (pSAG1::CAS9-U6::sgPuf2) using Q5 Hot Start site-directed mutagenesis kit (NEB), and primers (Table S1) to induce double-strand DNA breaks and facilitate the insertion of the PCR fragment. The PCR fragment containing the DHFR resistance cassette (DHFR-TS) was amplified from the plasmid pU6-SAG1-DHFR (Addgene #80322), flanking 30 bp homology with the 5′ and 3′ ends of TgPuf1 and TgPuf2 guide RNA targeting sites (89). 10 μg each of pSAG1::Cas9-U6::sgPuf1 or pSAG1::Cas9-U6::sgPuf2 and DHFR-TS amplicon were transfected into 10^7^ RHΔ*ku80* or ME49 or RH-TIR1 parasites by electroporation. Transfected parasites were then selected using 1 μM pyrimethamine and subsequently cloned. The insertion of the DHFR-TS cassette was confirmed through sequencing and diagnostic PCR. The knockout of the TgPuf1 (RH/ME49-*Δpuf1*) and TgPuf2 (RH/ME49/RH-TIR1-*Δpuf2*) genes was validated by immunoblotting and IF staining.

### Intracellular replication assay

HFF monolayers on glass coverslips in a 6-well plate were inoculated with freshly egressed 10^3^ RHΔ*ku80,* RHΔ*Puf1*, RHΔ*Puf2*, ME49, ME49Δ*puf1*, or ME49Δ*puf2* parasites and incubated for 24 h. Cells were then fixed and permeabilized in PBS containing 4% paraformaldehyde and 0.05% Triton X-100 and subsequently stained with α-TgIMC1 (1:2000/PBS) and DAPI to detect individual parasites. The number of parasites per vacuole was determined by counting the parasites in 50 random vacuoles for each experiment (n = 3).

### Plaque assay

Freshly egressed 100 RHΔ*ku80*, RHΔ*puf1*, or RHΔ*puf2* parasites were inoculated onto HFF monolayers in a 6-well plate and incubated for 5 days (95). Cells were then fixed with 100% ice-cold methanol for 20 min and stained with a 2% crystal violet solution for 20 min. Plaques, indicated by zones of lysis (white), were visualized against intact cells (purple). The number of plaques was calculated by observing 50 random fields for each experiment (n = 3). The plaque areas for RHΔ*puf1* and RHΔ*puf2* were quantified using ImageJ.

### Invasion assay

Freshly egressed 10^3^ RHΔ*ku80*, RHΔ*puf1*, RHΔ*puf2* parasites were allowed to infect HFFs grown on glass coverslips in a 6-well plate for 30 min at 37°C and then fixed with 4% paraformaldehyde (102). A standard non-permeabilizing IFA was performed by staining extracellular parasites with rabbit α-TgIMC1 antibodies (1:2000/PBS), followed by permeabilization with PBS containing 0.05% Triton X-100. Cells were then stained with mouse α-TgIMC1 antibodies (1:2000/PBS) to visualize invaded parasites. Subsequently, cells were stained with AF-conjugated secondary antibodies (anti-mouse IgG AF 488 and anti-rabbit IgG AF 594) and DAPI. For each experiment (n = 3), 100 parasites (green/red) were counted, distinguishing between non-invaded (red) and invaded (green) parasites.

To assess the invasion ability of RHΔ*ku80*, RHΔ*puf1*, and RHΔ*puf2* parasites under various physiological conditions, 10^3^ parasites from each strain were incubated for 1 h in 1 ml parasite medium containing high [K^+^], at pH 8.5, heat shock at 43 °C, or nutrient starvation. Subsequently, parasites were allowed to infect HFFs, and their invasion abilities were evaluated as described above.

### Egress assay

Freshly egressed 10^3^ RH*Δku80*, RH*Δpuf1* and RH*Δpuf2* parasites were inoculated on HFF monolayers grown on glass coverslips in a 6-well plate (95). After 30 h, parasite egress was initiated by adding 3 μM calcium ionophore A23187 to the medium and incubating for 2 min. Cells were then fixed, permeabilized, and stained with α-TgGRA2 (1:1000/PBS) and α-TgIMC1 (1:2000/PBS). Hundred vacuoles from each experiment (n = 3) were examined to count the intact or collapsed vacuoles. Vacuoles containing > 2 parasites were considered intact.

### Mouse infection

To assess whether TgPuf1 and TgPuf2 are essential for the tachyzoite stage, 100 tachyzoites of RHΔ*ku80*, RHΔ*puf1*, or RHΔ*puf2* strains were intraperitoneally injected into 6-week-old male BALB/c mice (n = 10/strain) and monitored daily for clinical signs and mortality.

To determine whether TgPuf1 and TgPuf2 are essential for the bradyzoite stage, 500 tachyzoites of ME49, ME49Δ*puf1*, or ME49Δ*puf2* strains were intraperitoneally injected into 6-week-old male BALB/c mice (n = 10/strain) and monitored daily for clinical signs and mortality for 50 days. All surviving mice were sacrificed by CO_2_ asphyxiation on day 50, and their brains were harvested to examine the cyst burden. Briefly, brains were dissected into 2 ml PBS and homogenized by repeated extruding through 23 and 18-gauge needles (12). An additional 5 ml of PBS was added, and homogenates were centrifuged at 1000 × *g* for 5 min. The supernatant was discarded, and the homogenate was resuspended in 1 ml PBS. A 20 μl brain homogenate was smeared on three glass slides and processed for immunofluorescence using CST1, a marker protein for cyst wall, to enumerate the cysts. The number of cysts per brain was determined based on CST1 positivity, and the mean number of cysts was calculated. The number of cysts per mouse was calculated as the average cyst number/slide ×50 (scaling factor) (103).

### Combined RNA fluorescent *in situ* hybridization (FISH), immunofluorescence

Freshly egressed parasites were collected and incubated for 1 h in parasite medium under various physiological conditions, including high [K^+^], a pH of 8.5, heat shock at 43 °C, and nutrient starvation. After incubation, the parasites were centrifuged and washed with DEPC-treated PBS, then smeared onto RNase-free glass slides. Next, parasites were fixed and permeabilized in DEPC-treated PBS containing 4% paraformaldehyde and 0.05% Triton X-100. The remaining protocol followed the methodology described by Thompson (2002) (7, 56, 104, 56, 104). Subsequently, 2× SSC buffer was added to the adhered parasites on the coverslips and incubated for 10 min as a pre-hybridization process. For the hybridization step, 2× SSC hybridization buffer containing 1 μM oligo dT, 10% dextran sulfate, 2 mM vanadyl-ribonucleoside complex, 0.02% RNAse-free BSA, 1 μg *E coli* tRNA, and 10% formamide. Further, the slides were incubated overnight in a humidified chamber at 37 °C for 16 h, followed by two washes with 2× SSC for 10 min each and one wash with 0.5× SSC for 5 min. Finally, these slides were processed for IFA as mentioned in the IF method with primary α-myc antibodies (1:100/PBS) and α-rabbit IgG secondary antibodies (1:1000/PBS).

### TUNEL assay

To evaluate parasite death under various physiological conditions, 10^3^ parasites from the RHΔ*ku80*, RHΔ*puf1*, and RHΔ*puf2* strains were incubated for 1 h in 1 ml of parasite medium with high [K^+^], at pH 8.5, heat shock at 43 °C, or nutrient starvation. Parasites were centrifuged and washed in PBS and then smeared onto glass slides. DNA fragmentation was detected using the One-Step TUNEL In Situ Apoptosis Kit (Elab Science #E-CK-A321), which utilizes immunofluorescence-based terminal deoxynucleotidyl transferase (TdT) dUTP nick end labelling (TUNEL) method (102). Briefly, cells were fixed, permeabilized, and equilibrated with TdT buffer at 37 °C for 30 min, followed by staining with TdT enzyme labeling solution for 60 min. The fluorescein signal was counterstained with DAPI. For the positive control, cells were treated with DNase I enzyme prior to the equilibration step. In each experiment (n = 3), 100 parasites were counted.

### Trypan blue staining

To evaluate parasite viability under various physiological conditions, 10^3^ parasites from the RHΔ*ku80*, RHΔ*puf1*, and RHΔ*puf2* strains were incubated for 1 h in 1 ml of parasite medium with high [K^+^], at pH of 8.5, heat shock at 43 °C, or nutrient starvation. After centrifugation and washing with PBS, the parasites were resuspended in 100 μl PBS and mixed with 100 μl of 0.4% trypan blue solution (95). After a 3-min incubation, parasites were counted in a Neubauer chamber using a light microscope with a 40× objective lens. In each experiment (n = 3), 100 parasites were counted, and viability was determined as the percentage of dead tachyzoites (trypan blue positive) relative to the total counted.

### Determination of gene expression (luminescence measurement and RT-qPCR)

The firefly luciferase gene (FLuc) was PCR amplified from the pmirGLO plasmid and cloned into the pTUB1:YFP-mAID-3HA, DHFR-TS:HXGPRT (Addgene # 87259) plasmid by replacing the YFP-mAID-3HA sequence at the *Bgl*II-*Nde*I sites. Similarly, the Renilla luciferase gene (RLuc), Renilla with wild-type PRE sequence (3xUGUAUAUA), and Renilla with mutant PRE sequence (3xUCCAUAUA) were PCR-amplified from pmirGLO and cloned into the same plasmid, replacing the YFP-mAID-3HA sequence at the *Bgl*II-*Nde*I sites. RHΔ*ku80*, RHΔ*puf1*, or RHΔ*puf2* tachyzoites (10^7^) were cotransfected with (2:1) 15 μg of RLuc or RLuc containing PRE (WT) or RLuc containing PRE (mut) and FLuc (7.5 μg) plasmid DNA. Immediately after transfection, parasites were inoculated on HFF cells, and after 24 h of infection, parasites were collected for luminescence measurement and RNA isolation. Luminescence was measured for the firefly and Renilla luciferase activities using the Dual-Luciferase Reporter Assay System (Promega, #E1910) by the EnSpire Multimode Plate Reader System. Firefly relative luminescence values were used for the normalization. Total RNA was isolated from these parasites using the RNeasy Plus mini kit (Qiagen), followed by cDNA synthesis using PrimeScript first strand cDNA Synthesis Kit (Takara). Specifically, 1 μg of RNA was mixed with oligo(dT), dNTPs, and PrimeScript reverse transcriptase, incubated at 30 °C for 10 min and 42 °C for 60 min, and finally heated at 95 °C for 5 min to inactivate the enzyme. qPCR was performed on the Bio-Rad CFX96 Real-Time PCR System using cDNA, gene-specific primers (Table S1), and SYBR Green PCR Master Mix (Applied Biosystems). qPCR reactions were performed using 10 ng cDNA with the following conditions: 1. 50 °C for 2 min, 2. 95 °C for 10 min, 3. 95 °C for 10 s, and 4. 60 °C for 35 s. Steps 3 and 4 were repeated for a total of 40 cycles. A melting curve analysis was conducted from 65 °C to 95 °C. For each run, no template control and no reverse transcriptase reactions were used to check for reagent contamination. Ct values were analyzed using the comparative Ct method (105, 106). Gene expression data were normalized to FLuc luciferase mRNA. ΔCt was calculated as ΔCt = Ct, RLuc (variants) - Ct, FLuc. ΔΔCt was determined by ΔΔCt = ΔCt, RLuc PRE (WT)/RLuc PRE (mut) - ΔCt, RLuc. The fold change (2ˆ−ΔΔCt) was used to determine differences in mRNA levels. Similar (as mentioned above) RNA isolation and RT-qPCR procedures were used to determine mRNA levels of PRE-containing genes in RHΔ*ku80*, RHΔ*puf1*, and RHΔ*puf2* parasites.

### Generation of auxin-inducible TgPuf1-mAID-3HA transgenic parasites

TgPuf1-mAID-3HA parasites were generated by CRISPR/Cas9-mediated site-specific gene editing (89, 107, 107) using the RHΔ*puf2*-TIR1 strain of *T*. *gondii.* This was accomplished by mutating the UPRT sequence from the pSAG1::Cas9-U6::sgUPRT plasmid (#54467, Addgene) using a Q5 Hot Start site-directed mutagenesis kit (#E0554S, NEB) and with specific primers (Table S1). The process aimed to induce double-strand DNA breaks and facilitate the insertion of the PCR fragment. The PCR fragment containing the mAID-3HA tag and the HXGPRT selection marker was amplified from the plasmid pTUB1:YFP-mAID-3HA (#87259, Addgene), with 40 bp of homology to the 3′ end of TgPuf1 to facilitate insertion through double homologous recombination. RHΔ*puf2* TIR1 strain parasites were cotransfected with 10 μg each of the TgPuf1-mAID-3HA amplicon and the pSAG1::Cas9-U6::sgPuf1 plasmid by electroporation using the Gene Pulser Xcell Total System (#1652660, BioRad). Transfected parasites were drug-selected with mycophenolic acid (25 μg/ml) and xanthine (50 μg/ml) for 3 growth cycles before cloning by serial dilution. Endogenous tagging of mAID-HA (TgPuf1-mAID-HA) in RHΔ*puf2*-TIR1 parasites was verified using immunoblotting and IF staining.

The auxin-induced degradation of TgPuf1-mAID-HA (RH-TIR1Δ*puf2* Puf1-mAID-HA) was assessed by culturing the parasites in medium containing 500 μM indole-3-acetic acid (IAA) (#I2886, Sigma) or MeOH for 2 h, followed by immunoblotting and IF staining with an α-HA antibody (#H3663, Merck Millipore). For replication assays, 10^3^ RH-TIR1, RH-TIR1Δ*puf2*, or RH-TIR1Δ*puf2* Puf1-mAID-HA parasites were seeded per well and incubated for 12 h, followed by treatment with 500 μM IAA or MeOH vehicle for 18 h. Parasite replication was assessed by IFA using α-TgIMC1 antibodies and by counting parasites in 50 randomly selected vacuoles per experiment (n = 3). The effect of TgPuf1 depletion on parasite multiplication was tested by culturing the parasites in a medium containing IAA or MeOH for 5 or 10 days (without changing the culture medium during this period), followed by staining with 2% crystal violet for plaque visualization, and plaque quantification was performed as described previously.

For the bradyzoite induction experiment, we cultured HFF-infected RH-TIR1Δ*puf2* Puf1-mAID-HA tachyzoites for an additional 4 days after 24 h of infection. The cultures were maintained in either bradyzoite induction medium, as previously described, or in DMEM containing IAA under standard conditions. The conversion to the bradyzoite stage was assessed at specified time points using IFA with an anti-TgCST1 antibody.

### Data analyses

All data analyses, including graph preparation and statistical evaluations, were performed using GraphPad Prism 9.

## Data availability

All data are included in this publication or in the supporting information, including figures, and tables. If additional information is desired, please contact Abhijit S. Deshmukh at abhijit@niab.org.in.

## Supporting information

This article contains supporting information.

## Conflict of interest

The authors declare that they have no conflicts of interest with the contents of this article.
