# Supplementary material for: PUF family RNA-binding proteins Puf1 and Puf2 promote transcript degradation in Toxoplasma gondii
Source: J Biol Chem. 2026 Aug 12;302(10):113436. doi: 10.1016/j.jbc.2026.113436 (PMC13599632; doi:10.1016/j.jbc.2026.113436)
Supplement: Supplementary file 1 [file mmc1.docx]

**Supplementary Fig: 1**

A	B

Supplementary Fig:1,(A-B) A phylogenetic tree of canonical Puf proteins was constructed by using MEGA X software with maximum likelihood analysis of TgPuf1 (A), and TgPuf2 (B), after aligning full-length protein sequences in 500 bootstrap replicates. The empty square represents 200 -500a.a, filled square 500-1000 a.a, empty circle represents 1000-1500 a.a, filled circle 1500-2000 a.a, and empty triangle represent >2000 a.a.

**Supplementary Fig: 2**

A	B

Supplementary Fig:2 (A-B), A phylogenetic tree of non-canonical proteins were constructed by using MEGA X software with maximum likelihood analysis of TgPum3 (A), and TgNop9 (B), after aligning full-length protein sequences in 500 bootstrap replicates. The empty square represents 200 -500a.a, filled square 500-1000 a.a, empty circle represents 1000-1500 a.a, and filled circle 1500-2000 a.a,

**Supplementary Fig: 3**

Supplementary Fig:3, Expression levels of TgPuf1 (A) and TgPuf2 (B) in tachyzoites (RH and ME49) and bradyzoites (ME49) were assessed using α-TgPuf1 and α-TgPuf2 antibodies. Loading controls: TgSAG1 - tachyzoite stage marker, and TgBAG1 - bradyzoite stage marker.

**Supplementary Fig: 4**

Supplementary Fig:4, The expression levels of TgPuf1 in RH and RH∆*puf2* tachyzoites using α-TgPuf1 antibodies and vice versa. Expression levels were normalized to TgALD.

**Supplementary Fig: 5**

Supplementary Fig:5, The expression levels of TgPuf1 in ME49 and ME49∆*puf2* tachyzoites using α-TgPuf1 antibodies and vice versa. Expression levels were normalized to TgALD.

**Supplementary Fig: 6**

Supplementary Fig.6: TgPuf1 and TgPuf2, full-length structural predictions using ColabFold v1.5.5. (A) The predicted structure of TgPuf1 (1676 aa) PAE indicates a low confidence score. (B) The predicted structure of TgPuf2 (1912 aa) PAE indicates a low confidence score. A Poor PAE score shows the low complexity of the unstructured regions.

**Supplementary Fig: 7**

Supplementary Fig: 7. Immunoblot analysis showing the expression of TgPABP in RH parasites detected using HsPABP antibody.

**Supplementary Fig: 8**

Supplementary Fig.8: Phylogenetic tree of TgPop2 protein was constructed by using MEGA X software with maximum likelihood analysis, after aligning full-length protein sequences in 500 bootstrap replicates. The empty square represents 200 -500a.a, filled square 500-1000 a.a, empty circle represents 1000-1500 a.a, filled circle 1500-2000 a.a, and empty triangle represent >2000 a.a.

**Supplementary Fig: 9**

**A.**

**B.**

```
ScPop2   MQSMNVQPRVLAVGGEQFFSQRQASEQHQQQNMGPQVYSPKVNRARMFPQGMPVNTINGS	60
TgPop2   ------------------------------------------------------------	0
HsPop2   ------------------------------------------------------------	0
AtPop2   ------------------------------------------------------------	0

ScPop2   VNQEMNNAYLLKQKNEPLLTQQQQQQQQQQQPFNIGTPVSVASLPPGLNVLQQQQQQQQQ	120
TgPop2   ------------------------------------------------------------	0
HsPop2   ------------------------------------------------------------	0
AtPop2   ------------------------------------------------------------	0

ScPop2   QQQQQQGVGLNRPLASQLPKHLTNQSMPPIFLPPPNYLFVRDVWKSNLYSEFAVIRQLVS	180
TgPop2   ---------------------------MN--GECGEREQIVEVWEHNLEEEFARIRDVVE	31
HsPop2   --------------------------MPA--ALVENSQVICEVWASNLEEEMRKIREIVL	32
AtPop2   --------------------------MSL--FLKDDSIQIREVWNDNLQEEMDLIRDVVD	32
                                            :   : :**  ** .*:  **::*
ScPop2   QYNHVSISTEFVGTLARPIGTFRSKVDYHYQTMRANVDFLNPIQLGLSLSDANGNKPDNG	240
TgPop2   RFQYIAMDTEFPGIVARPTGNV---TDYNYQTVKYNVDLLKVIQLGITFADADGNLAEG-	87
HsPop2   SYSYIAMDTEFPGVVVRPIGEFRSSIDYQYQLLRCNVDLLKIIQLGLTFTNEKGEYPSG-	91
AtPop2   DFPYVAMDTEFPGIVVRPVGTFKSNADYHYETLKTNVNILKMIQLGLTFSNEQGNLPTCG	92
          : ::::.*** * :.** * .    **:*: :: **::*: ****::::: .*:

ScPop2   ---PSTWQFNFE-FDPKKEIMSTESLELLRKSGINFEKHENLGIDVFEFSQLLMDSGLMM	296
TgPop2   ---TSTWQFNFR-FDLNEDMYAQDSIDFLKQSGIDFDKQQKKGIDVQDFGELIMNSGLVM	143
HsPop2   ---INTWQFNFK-FNLTEDMYSQDSIDLLANSGLQFQKHEEEGIDTLHFAELLMTSGVVL	147
AtPop2   TDKYCIWQFNFREFDLDSDIFALDSIELLKQSGIDLAKNTLDGIDSKRFAELLMSSGIVL	152
               *****. *:  .:: : :*:::* :**::: *:   ***   *.:*:* **:::

ScPop2   DDSVTWITYHAAYDLGFLINILMNDSMPNNKEDFEWWVHQYMPNFYDLNLVYKIIQEFKN	356
TgPop2   NEDVKWISFHGCYDFGYLLKLLTCAPLPHSEAQFFELLHDFFPSLYDIKYLLRSIHNFNL	203
HsPop2   CDNVKWLSFHSGYDFGYMVKLLTDSRLPEEEHEFFHILNLFFPSIYDVKYLMKSCKNLK-	206
AtPop2   NENVHWVTFHSGYDFGYLLKLLTCQNLPDSQTDFFKLINVYFPTVYDIKHLMKFCNSLH-	211
          :.* *:::*. **:*:::::*    :*..: :*   :: ::*..**:: : :  :.::

ScPop2   PQLQQSSQQQQQQQYSLTTLADELGLPRFSIFTTTGGQSLLMLLSFCQLSKLSMHKFPNG	416
TgPop2   S-----------GGCSLQKIAEHLQVTRVGPQHQAGSDSLVTCRTFF---KLVELYFDSS	249
HsPop2   --------------GGLQEVADQLDLQRIGRQHQAGSDSLLTGMAFF---RMKELFFEDS	249
AtPop2   --------------GGLNKLAELLEVERVGICHQAGSDSLLTSCTFR---KLKENFFVGP	254
                        .*  :*: * : *..    :*.:**:   :*    ::    * .

ScPop2   TDFAKYQGVIYGIDGDQ-------------------------------------------	433
TgPop2   IDDCGYSGVIYGLGMSIPKRGGSNQHLPPVSSRSSDSLGRGARPGKTRAPNDDGDDTAGS	309
HsPop2   IDDAKYCGRLYGLGTGVAQKQNEDVDSA--QEKMS-ILAIIN---NMQQ-----------	292
AtPop2   L--HKYSGVLYGLGVENEANGKN----V--SYRMD-MVAWA-------------------	286
                 * * :**:.

ScPop2   ------------------------------------------------------------	433
TgPop2   GASNAATSAAPGRMLSGTSSSSFSGVGTGGGAAAQAGMTHGNHVPFHAGHSAQPGAGHHL	369
HsPop2   ------------------------------------------------------------	292
AtPop2   ------------------------------------------------------------	286

ScPop2   ------------------------------------------------------------	433
TgPop2   LNAHPLSFSGSSGPSASTPEFVPAALANNLHASQHLPLSNASLSLLAAPNPDMAATHGSF	429
HsPop2   ------------------------------------------------------------	292
AtPop2   ------------------------------------------------------------	286

ScPop2   ------------------------------------------------------------	433
TgPop2   LLSAAGPQGAPASTLHSKASRQVLGASGAAPGTSSATHLRSQRGNLAAGLLSGSGGPQLF	489
HsPop2   ------------------------------------------------------------	292
AtPop2   ------------------------------------------------------------	286

ScPop2   ------------------------------------------------------------	433
TgPop2   GLQTAVPTAVAAGPGDSAGGPQGTHAENAFEAGRGALRLPLAGGTRESCGRSASFLGQER	549
HsPop2   ------------------------------------------------------------	292
AtPop2   ------------------------------------------------------------	286

ScPop2   ------------------------------------------------------------	433
TgPop2   DRSQATQGLAGSGLLVLQQVANGGVGSGEGAETAGAKSVGTFGGNLWGGANAGEEERKKS	609
HsPop2   ------------------------------------------------------------	292
AtPop2   ------------------------------------------------------------	286

ScPop2   --------	433
TgPop2   GTGTNAQE	617
HsPop2   --------	292
AtPop2   --------	286
```

Supplementary Fig.9: (A) The TgPop2 protein contains an N-terminal CAF1 ribonuclease-like domain, which spans amino acids 11 to 251, and a C-terminal low-complexity region. (B) TgPop2 full length sequence analysis. An alignment of the amino acid sequence of TgPop2 with *S*. *cerevisiae* (ScPop2), *H*. *sapiens* (HsPop2), and *A*. *thaliana* (AtPop2) revealed the presence of conserved caf1 ribonuclease domain. The highly conserved amino acid residues (D39, E41, D157, and D230) within the domain are highlighted.

**Supplementary Fig: 10**

**A.**

**B.**

**C.**

|  | **Helix** | **Antiparallel** | **Parallel** |
| --- | --- | --- | --- |
| TgPop2 | 55.9% | 22.1% | 22.1% |
| TgPop2E41A | 50.1% | 22.8% | 26.8% |

Supplementary Fig.10: (A) Chromatogram showing point mutation replacing TgPop2E41A. (B) Purification profile of TgPop2 and TgPop2E41A (~64kDa) analysed on 10% SDS-PAGE. (C) Circular Dichroism (CD) spectroscopy of TgPop2 and TgPop2 (E41A). Proteins were diluted to 0.3 mg/ml, and far-UV CD spectra were recorded in triplicates over the range of 190-340 nm at 4 °C using a Jasco CD spectrophotometer. The resulting spectra were analysed to estimate the secondary structure content using BeStSel tool.

**Supplementary Fig: 11**

Supplementary Fig.11: Release of AMP measured from the Poly(A)20 RNA substrate in the presence of increasing concentrations of TgPop2 protein (0-500 nM) using Amp Glo assay kit.

**Supplementary Fig: 12**

Supplementary Fig.12: Exonucleolytic activity of TgPop2 was analyzed using Thin-Layer Chromatography (TLC). TgPop2 releases α-^32^P-AMP from an RNA substrate that ends with a poly(A) tail, where the RNA is internally labeled with α-^32^P at the adenosine residues.

**Supplementary Fig : 13**

Supplementary Fig.13: *In vitro* deadenylation assays were conducted using recombinant TgPuf1/2 and TgPop2 proteins. After the specified incubation periods, the reaction products were separated using denaturing polyacrylamide gel electrophoresis. The PRE substrate RNA, which contained 30 adenosines at its 3′ end, was internally labeled with α-^32^P. It was then incubated with 100 nM of TgPop2 protein for the indicated time, in minutes, either without (–) or with (+) the addition of purified recombinant TgPuf1/2.

**Methodology for Supplementary Figs: 12 and 13**

**i) RNA substrates**

RNA substrates were synthesized using a DNA template that featured the T3 promoter and a PUM response element (PRE) sequence, followed by 30 adenine (polyA) bases. In vitro transcription of this PRE-containing DNA template was performed using T3 polymerase, incorporating α-^32^P-ATP for internal labeling of the RNA. The internally radiolabeled PRE RNA substrate was then utilized for thin-layer chromatography (TLC) analysis.

**ii) Thin layer chromatography (TLC)**

The radiolabelled PRE RNA substrates were incubated with different concentrations of TgPop2 protein for 30 min at 30°C in a buffer containing 40 mM Tris (pH 8.0), 100 mM NaCl, 2 mM MgCl2, 5% (v/v) glycerol, and 2 mM DTT. Samples from the exonuclease reaction with internally radiolabelled RNA substrates were taken at the indicated time points and separated on PEI TLC cellulose plate (MN) in 0.1 M sodium phosphate buffer, pH 6.8. Following separation, TLC plates were dried and visualized with the Typhoon Phosphor Imager.

**iii) Deadenylation assay**

Exonuclease activity reactions (20 µL) were conducted at 30°C in a buffer solution containing 40 mM Tris (pH 8.0), 100 mM NaCl, 2 mM MgCl₂, 5% (v/v) glycerol, and 2 mM DTT. The reaction mixture was incubated with specific concentrations of TgPop2, either alone or in combination with TgPuf1 and TgPuf2, for 10 or 20 minutes. Reactions were initiated by adding uniformly radiolabeled α-^32^P-ATP RNA substrate to achieve the desired final concentrations. At predetermined time intervals, aliquots were taken, and the reactions were stopped by adding an equal volume of denaturing loading buffer (comprising 80% formamide, 0.025% [w/v] xylene cyanol, and bromophenol blue). The samples were then heated at 95°C, loaded onto a 20% denaturing polyacrylamide gel (acrylamide: bis 19:1, 7 M urea), and resolved to achieve single-nucleotide resolution. After separation, the gels were desiccated, and the individual RNA species were visualized using a Typhoon Phosphor Imager.
