# Supplementary material for: PUF family RNA-binding proteins Puf1 and Puf2 promote transcript degradation in Toxoplasma gondii
Source: J Biol Chem. 2026 Aug 12;302(10):113436. doi: 10.1016/j.jbc.2026.113436 (PMC13599632; doi:10.1016/j.jbc.2026.113436)
Supplement: Supplementary file 2 [file mmc2.docx]

**Supplementary Table 1**

**List of primers and oligos**

| **Sr. No.** | **Name** | **Sequence** | **Type** |
| --- | --- | --- | --- |
| 1 | TgPuf1_LIC_F | 5’-TACTTCCAATCCAATTTAATGCAACGTCGTTGAAAAGGTAGAG-3’ | DNA transfection |
| 2 | TgPuf1_LIC_R | 5’- TCCTCCACTTCCAATTTTAGCCATCCCATCGACAGCAATCA-3’ | DNA transfection |
| 3 | TgPuf2_LIC_F | 5’-TACTTCCAATCCAATTTAATGCGCAAGCACATTGTGACGGC-3’ | DNA transfection |
| 4 | TgPuf2_LIC_R | 5’-TCCTCCACTTCCAATTTTAGCCGTCGGCCGACACTGTTG-3’ | DNA transfection |
| 5 | TgPuf1-RBD_F | 5’-GGAATTCCATATGGGCACCGATGCACATTT-3’ | Bacterial cloning |
| 6 | TgPuf1-RBD_R | 5’-CCCTCGAGTTAGTGGTGATGGT-3’ | Bacterial cloning |
| 7 | TgPuf2-RBD_F | 5’-GGAATTCCATATGGGTTTTTTTGTTGGTT-3’ | Bacterial cloning |
| 8 | TgPuf2-RBD_R | 5’-CCCTCGAGTTAATGATGATGGTG-3’ | Bacterial cloning |
| 9 | Renilla_F | 5’GAAGATCTATGGCTTCCAAGGTGTACGA-3' | DNA transfection |
| 10 | Renilla_R | 5’-GGAATTCCATATGTTATTACTGCTCGTTCTTCAGCACG-3’ | DNA transfection |
| 11 | Renilla_PRE-WT_F | 5’GAAGATCTATGGCTTCCAAGGTGTACGA-3' | DNA transfection |
| 12 | Renilla_PRE-WT_R | 5’-GGAATTCCATATGTATATACAATATATACAATATATACATTATTACTGCTCGTTCTTCAGCACG-3’ | DNA transfection |
| 13 | Renilla_PRE-mut_F | 5’GAAGATCTATGGCTTCCAAGGTGTACGA-3' | DNA transfection |
| 14 | Renilla_PRE-mut_R | 5’-GGAATTCCATATGTATAAGGTATATAAGGTATATAAGGTTTATTACTGCTCGTTCTTCAGCACG-3’ | DNA transfection |
| 15 | TgPuf1_sgRNA_F | 5’-GAGACTGGAAGAATTTCTGAGTTTTAGAGCTAGAAATAGC-3’ | DNA transfection |
| 16 | AID_Cas9_R | 5′-AACTTGACATCCCCATTTAC-3' | DNA transfection |
| 17 | TgPuf1_DHFR_F | 5’-CGGTTCGTGAAGTAAACGTGCACACCCGCAGCACTCCGGCATGCAGAAACCGGTG-3’ | DNA transfection |
| 18 | TgPuf1_DHFR_R | 5'-TGCCCCGGCGCGTATCCTGTTCAACGCAAACCTACCATTTCTAGACAGCCATCTC-3' | DNA transfection |
| 19 | TgPuf2_sgRNA_F | 5’-GACGCACTTGTGACTTTGCCGTTTTAGAGCTAGAAATAGC-3’ | DNA transfection |
| 20 | TgPuf2_DHFR_F | 5’-CTCTGCGGAGAGAAAAGACCGCGTGGGTTTGGAGCCCGGAATGCAGAAACCGGTG-3’ | DNA transfection |
| 21 | TgPuf2_DHFR_R | 5'-GCCGGCATCTGTCTCTTCACGTTGCTGGGGGTCGGAGAATCTAGACAGCCATCTC-3' | DNA transfection |
| 22 | TgPuf1_PCR1_F | 5'-TCGTGAAGTAAACGTGCACAC-3' | Diagnostic PCR |
| 23 | TgPuf1_PCR1_R | 5’-GGAGGAGATCATAAAAAAACTGTTGC-3’ | Diagnostic PCR |
| 24 | TgPuf1_PCR2_F | 5'-TCGTGAAGTAAACGTGCACAC-3' | Diagnostic PCR |
| 25 | TgPuf1_PCR2_R | 5’-GGCAACGCCCAGAGACAG-3’ | Diagnostic PCR |
| 26 | TgPuf2_PCR1_F | 5'-TCCCTCTGCGGAGAGAAAAG-3' | Diagnostic PCR |
| 27 | TgPuf2_PCR1_R | 5’-ATGCTGATGTTCGGCGCCC-3’ | Diagnostic PCR |
| 28 | TgPuf2_PCR2_F | 5'-TCCCTCTGCGGAGAGAAAAG-3' | Diagnostic PCR |
| 29 | TgPuf2_PCR2_R | 5’-GGCAACGCCCAGAGACAG-3’ | Diagnostic PCR |
| 30 | FLuc_F | 5’-CCAGCCAGGTTACAACCGC-3' | RT-qPCR |
| 31 | FLuc_R | 5’-CACGGCGATCTTGCCGCC-3’ | RT-qPCR |
| 32 | RLuc_F | 5’-CCTAAGATGTTCATCGAGTCC-3’ | RT-qPCR |
| 33 | RLuc_R | 5’-CTTGATGTACTTACCCATTTCA-3’ | RT-qPCR |
| 34 | DM NRE | 5'Cy5-AUUAUUUUGUUGUCGAAAAUUGUACAUAAGCC | MST experiment |
| 35 | TgPRE WT | 5'Cy5- CAGACAAUUUGCGACAACACGCAUGUAUAUAUAGU | MST experiment |
| 36 | TgPRE mut | 5'Cy5- CAGACAAUUUGCGACAACACGCAUCCAUAUAUAGU | MST experiment |
| 37 | PRE-RNA (WT) | 5’CAGACCUGUAUAUACACACUAAAAAAAAAAAAAAAAAAAA | AMP release |
| 38 | PRE-RNA (mut) | 5’CAGACCUCCAUAUACACACUAAAAAAAAAAAAAAAAAAAA | AMP release |
| 39 | TgClp_F | 5’-GAGGAGAGAGCGGGAGGAA-3’ | RT-qPCR |
| 40 | TgClp_R | 5’- ACTGCGTCCACGAGGCCG-3 | RT-qPCR |
| 41 | TgNADH1_F | 5’-GACGGCGCAGAACGCGAA-3’ | RT-qPCR |
| 42 | TgNADH1_R | 5’-TCTGCAGCTGCTTGCCCAT-3’ | RT-qPCR |
| 43 | TgACP12_F | 5’- CAGCAACACTACTCCGGTG -3’ | RT-qPCR |
| 44 | TgACP12_R | 5’- CAGACATAGACATTTGCATGAC-3’ | RT-qPCR |
| 45 | TgDRPA_F | 5’- GCGAGCAGATCGAAACAG -3’ | RT-qPCR |
| 46 | TgDRPA_R | 5’- CAACGAGCTCGCGCTGTA -3’ | RT-qPCR |
| 47 | TgCdpk2_F | 5’- GGAAAGTCGCCGACGCTC -3’ | RT-qPCR |
| 48 | TgCdpk2_R | 5’- CTTCGATAGGCGGTTGTC -3’ | RT-qPCR |
| 49 | TgGNAT_F | 5’-GCATGCGGATCATGAACGA-3’ | RT-qPCR |
| 50 | TgGNAT_R | 5’- CGACTCTAACGAAAGAGAG-3’ | RT-qPCR |
| 51 | TgGAPDH_F | 5’-CTGAACGGCAAGCTGACCG-3’ | RT-qPCR |
| 52 | TgGAPDH_R | 5’- TCCCTTCATGGGGCCGGA-3’ | RT-qPCR |
| 53 | TgPop2_E41A_F | 5’-ATCGCGATGGACACCGCGTTTCCGGGTATTGTG-3’ | SDM |
| 54 | TgPop2_E41A_R | 5’- CACAATACCCGGAAACGCGGTGTCCATCGCGAT-3’ | SDM |
| 55 | TgPuf1_AID_F | 5'-AGATCGACAAAAGACGTGATGTTTTAGAGCTAGAAATAGC-3’ | sgRNA |
| 56 | TgPuf1_MH_F | 5'-GACGGACACCAGCCCGGCAATGATTGCTGTCGATGGGATGGCTAGCAAGGGCTCGGGC-3' | Micro-homology |
| 57 | TgPuf1_MH_R | 5'-ATTGCGTGAAGTCCAACAAACGTGGGCTTTTACCGTAAACATAGGGCGAATTGGAGCTCC-3' | Micro-homology |
| 58 | TgPop2-GST_F | 5’-CGGATCCATGAATGGCGAGTGCGGCG-3’ | Bacterial cloning |
| 59 | TgPop2-GST_R | 5’-CCCTCGAGTTACTCCTGCGCGTTGGCC-3’ | Bacterial cloning |
