# Supplementary material for: PUF family RNA-binding proteins Puf1 and Puf2 promote transcript degradation in Toxoplasma gondii
Source: J Biol Chem. 2026 Aug 12;302(10):113436. doi: 10.1016/j.jbc.2026.113436 (PMC13599632; doi:10.1016/j.jbc.2026.113436)
Supplement: Supplementary file 3 [file mmc3.docx]

**Supplementary Table 2**

List of high-confidence PRE containing genes (n=146) in *T*. *gondii*.

| **Gene_ID**  **TGME49#** | **Gene Name** | **LOPIT** | **Motif_**  **count** | **UTR** | **CRISPR** |
| --- | --- | --- | --- | --- | --- |
| 209430 | 40S ribosomal protein S4, putative | Nucleolus | 3 | 5' | -4.72 |
| 261850 | plastid replication-repair enzyme PREX | Apicoplast | 3 | 5' | -5.23 |
| 267800 | dynamin-related protein DRPA | Nucleus - chromatin | 4 | 5' | -4.1 |
| 289350 | ATP-binding cassette G family transporter ABCG84 | PM - integral, Golgi | 2 | 5' | 0.44 |
| 294430 | protein SDA1, putative | nucleus - chromatin | 3 | 5' | -3.87 |
| 202790 | dihydrouridine synthase (dus) protein | NA | 2 | 3' | -0.81 |
| 203270 | ATP-dependent Clp endopeptidase | NA | 2 | 3' | -3.81 |
| 204160 | GYF domain-containing protein | Nucleus- chromatin, non chromatin | 2 | 3' | -4.57 |
| 205625 | transporter, major facilitator family protein | Golgi | 2 | 3' | -2.04 |
| 208718 | protein tyrosine phosphatase type IVA A, putative | NA | 2 | 3' | -2.3 |
| 209150 | non-proton pumping type-II NADH dehydrogenase I | Mitochondrion - membranes | 3 | 3' | -0.13 |
| 209280 | FAM76 domain-containing protein | Nucleus- chromatin, non chromatin | 3 | 3' | -0.2 |
| 210390 | WD domain, G-beta repeat-containing protein | NA | 3 | 3' | -1.58 |
| 213840 | choline transporter-like protein, putative | NA | 2 | 3' | -0.25 |
| 214090 | signal peptidase | Nucleus - non-chromatin | 3 | 3' | -0.3 |
| 214490 | peptidase M16 inactive domain-containing protein | Cytosol, 19S proteasome | 2 | 3' | -4.1 |
| 215250 | thiamin pyrophosphokinase, catalytic domain-containing protein | mitochondrion - soluble | 3 | 3' | -3.28 |
| 216530 | ribosome recycling factor protein | Mitochondria | 5 | 3' | -3.81 |
| 216970 | coronin, putative | NA | 3 | 3' | 0.71 |
| 217020 | ATPase, AFG1 family protein | Mitochondria | 4 | 3' | 0.02 |
| 217160 | Der1 family protein | NA | 4 | 3' | -0.56 |
| 217870 | palmitoyltransferase DHHC3 | PM - integral | 3 | 3' | 0.47 |
| 223970 | translation elongation and release factors (gtpases), putative | Plamsa membrane - peripheral 2 | 2 | 3' | -4.23 |
| 225108 | SNF7 family protein | NA | 4 | 3' | -1.57 |
| 226068 | DnaJ domain-containing protein | Endoplasmic reticulum | 4 | 3' | -4.73 |
| 227780 | P-type ATPase, putative | Nucleolus | 4 | 3' | -1.38 |
| 230060 | acetyltransferase, GNAT family protein | Nucleolus | 2 | 3' | -0.04 |
| 230340 | apical annuli protein AAP4 | Apical 1 | 2 | 3' | -1.14 |
| 231370 | patatin-like phospholipase PL2 | Apicoplast | 6 | 3' | -4.05 |
| 233710 | inorganic anion transporter, sulfate permease (SulP) family protein | NA | 3 | 3' | -2.2 |
| 236080 | replication factor-a protein 1 (rpa1) subfamily protein | Nucleus- chromatin, non chromatin | 5 | 3' | -3.35 |
| 236240 | tyrosine kinase-like protein TKL6 | Nucleolus | 3 | 3' | -0.57 |
| 236510 | TMEM65 domain-containing protein | Mitochondra -Membrane | 3 | 3' | -2.74 |
| 236570 | lysine decarboxylase family protein | Cytosol | 2 | 3' | -3.75 |
| 237860 | calcium-dependent protein kinase CDPK7A | NA | 2 | 3' | -3.24 |
| 238130 | HXXEE motif protein | IMC | 3 | 3' | -0.47 |
| 238170 | IMC-associated protein IAP3 | NA | 2 | 3' | -5.06 |
| 238950 | stearoyl-CoA desaturase | Endoplasmic reticulum | 3 | 3' | -3.75 |
| 239490 | 2-oxoisovalerate dehydrogenase subunit alpha | Mitochondrion - soluble | 4 | 3' | -1.89 |
| 240210 | phosphate transporter family protein, putative | NA | 2 | 3' | -1.34 |
| 240860 | acyl-glycerol-3-phosphate acyltransferase AGPAT | Endoplasmic reticulum | 2 | 3' | -2.54 |
| 242000 | AMMECR1 domain-containing protein | Cytosol | 4 | 3' | 0.78 |
| 244200 | 2-oxoglutarate dehydrogenase e1 component, mitochondrial precursor, putative | Mitochondrion - soluble | 3 | 3' | -4.82 |
| 246600 | ABC1 family protein | Endoplasmic reticulum | 5 | 3' | -0.49 |
| 246670 | hypothetical protein | Nucleolus | 3 | 3' | -3.64 |
| 246750 | hypothetical protein | NA | 2 | 3' | -0.06 |
| 247370 | polycystin cation channel protein | N/A | 6 | 3' | -1.42 |
| 248110 | repressor of RNA polymerase III transcription MAF1, putative | Nucleolus | 4 | 3' | -0.1 |
| 249440 | apical cap protein AC12 | NA | 3 | 3' | -0.48 |
| 249702 | MC family transporter, putative | Mitochondria-Membrane | 3 | 3' | 0.28 |
| 249770 | Nmda1 protein | NA | 3 | 3' | 1.41 |
| 255430 | Rad9 protein | NA | 2 | 3' | 0 |
| 256820 | zinc finger (CCCH type) motif-containing protein | Nucleus - chromatin | 3 | 3' | -4.96 |
| 257290 | RNA 2'-phosphotransferase, Tpt1/KptA family protein | NA | 3 | 3' | -2.54 |
| 258000 | hypothetical protein | Mitochondrion - soluble | 2 | 3' | -3.53 |
| 258080 | golgi localizing protein GLP1 | Nucleolus | 3 | 3' | -2.38 |
| 259070 | acetyltransferase, GNAT family protein | N/A | 13 | 3' | -2.51 |
| 259830 | diacylglycerol kinase DGK2 | Dense granules | 4 | 3' | 0.05 |
| 260200 | helicase associated domain (ha2) protein | Nucleolus | 2 | 3' | -5.65 |
| 261370 | F-box domain-containing protein | NA | 2 | 3' | -2.9 |
| 261475 | Hypothetical protein | Mitochondrion-soluble | 2 | 3' | -4.09 |
| 261955 | hypothetical protein | NA | 3 | 3' | -3.75 |
| 262000 | AP2 domain transcription factor AP2VIIb-2 | MT | 2 | 3' | 0.61 |
| 262520 | cyclophilin, putative | Mitochondrion-soluble | 2 | 3' | -1.76 |
| 264820 | RbAp48 | Nucleus - chromatin | 3 | 3' | -1.31 |
| 265770 | apicomplexan kinetochore protein AKIT5, putative | NA | 3 | 3' | -5.01 |
| 270100 | oxidoreductase, aldo/keto reductase family protein | NA | 2 | 3' | -5.13 |
| 270830 | small nuclear ribonucleoprotein | Nucleus- chromatin, non chromatin | 3 | 3' | -2.22 |
| 271440 | nuclear protein localization protein 4, putative | Nucleolus | 6 | 3' | -4.16 |
| 273760 | heat shock protein HSP70 | Cytosol | 2 | 3' | -4.97 |
| 275490 | nucleoporin, putative | Nucleolus | 2 | 3' | -4.18 |
| 275840 | protein phosphatase PPM19, putative | Mitochondrion-soluble | 3 | 3' | -0.14 |
| 277500 | 26S proteasome regulatory subunit 7, putative | 19S proteasome | 5 | 3' | -3.35 |
| 278490 | Zn-finger, RING domain containing protein | Nucleus- chromatin, non chromatin | 5 | 3' | -0.01 |
| 280770 | regulator of chromosome condensation (RCC1) repeat-containing protein | NA | 2 | 3' | -4.59 |
| 281440 | hypothetical protein, DUF3712 domain-containing protein | Golgi | 6 | 3' | -4.83 |
| 282140 | cwf21 protein | Nucleus- chromatin, non chromatin | 4 | 3' | -3.44 |
| 285268 | DnaJ domain-containing protein | Cytosol | 3 | 3' | -4.17 |
| 286160 | non-specific serine/threonine protein kinase | Nucleolus | 4 | 3' | NA |
| 288210 | PUL domain-containing protein | Cytosol | 4 | 3' | -2.14 |
| 289490 | protein phosphatase PPM11A, putative | N/A | 10 | 3' | -2.46 |
| 289770 | AP-1 complex subunit mu-1, putative | Nucleus - chromatin | 3 | 3' | -5.03 |
| 290260 | thioredoxin family Trp26 protein | Cytosol | 2 | 3' | 0.42 |
| 290570 | Hypothetical protein | NA | 3 | 3' | -3.87 |
| 290850 | WD-40 repeat protein | Nucleus - non-chromatin | 2 | 3' | -4.5 |
| 293060 | SPRY domain-containing protein | Nucleus - non-chromatin | 4 | 3' | -0.18 |
| 293900 | sporozoite protein with an altered thrombospondin repeat SPATR | Micronemes | 3 | 3' | 0.1 |
| 294700 | polynucleotide 5'-hydroxyl-kinase | Nucleus- chromatin, non chromatin | 2 | 3' | -4.07 |
| 297500 | T-complex protein 1 eta subunit | Cytosol | 2 | 3' | -5.99 |
| 304680 | DNA damage-inducible protein 1 | Cytosol | 2 | 3' | -0.95 |
| 305020 | CS domain-containing protein | NA | 3 | 3' | -4.44 |
| 305150 | tetratricopeptide repeat-containing protein | NA | 2 | 3' | -1.57 |
| 307030 | Purine nucleoside phosphorylase | NA | 2 | 3' | 0.24 |
| 308000 | Gpi16 subunit, GPI transamidase component protein | Endoplasmic reticulum | 3 | 3' | -5.22 |
| 308093 | rhoptry kinase family protein (incomplete catalytic triad) | Rhoptries 1 | 1 | 3' | NA |
| 310860 | U5 snRNP-specific protein | Nucleus- chromatin, non chromatin | 5 | 3' | -0.51 |
| 311300 | cAMP-dependent protein kinase regulatory subunit 2, putative | NA | 3 | 3' | 1.08 |
| 311320 | Qb-SNARE protein, putative | NA | 2 | 3' | -3.41 |
| 313580 | cytochrome b5 family heme/steroid binding domain-containing protein | Nucleolus | 3 | 3' | -1.51 |
| 314470 | hypothetical protein | Nucleus- chromatin, non chromatin | 2 | 3' | -4.33 |
| 314860 | zinc knuckle domain-containing protein | Nucleus- chromatin, non chromatin | 5 | 3' | -1.53 |
| 315590 | macro domain-containing protein | NA | 3 | 3' | -0.42 |
| 315690 | DnaJ domain-containing protein | Nucleolus | 3 | 3' | -2.9 |
| 316700 | uridine kinase | NA | 3 | 3' | -2.4 |
| 316730 | non-discharge partner NDP2 | PM | 3 | 3' | -4.39 |
| 318190 | phosphoglycerate mutase family protein | NA | 2 | 3' | -1.05 |
| 318410 | TCP-1 chaperonin, putative | Cytosol | 4 | 3' | -5.13 |
| 319390 | hypothetical protein | NA | 5 | 3' | -0.34 |
| 319650 | 3' exoribonuclease family, domain 1 domain-containing protein | Nucleus- chromatin, non chromatin | 2 | 3' | -4.45 |
| 500134 | RNA binding protein, putative | NA | 3 | 3' | -1.02 |
| 500228 | 30S ribosomal protein S10, putative | NA | 3 | 3' | -4.03 |
| 500287 | phosphatidylinositol 4-kinase, putative | NA | 4 | 3' | -2.7 |
| 500363 | regulator of chromosome condensation, putative | NA | 3 | 3' | -0.61 |
| 202730 | hypothetical protein | NA | 6 | 5' | -3.47 |
| 214100 | hypothetical protein | NA | 3 | 5' | 1.72 |
| 231200 | hypothetical protein | NA | 9 | 5' | -4.01 |
| 257420 | hypothetical protein | NA | 3 | 5' | -1.31 |
| 257700 | hypothetical protein | NA | 3 | 5' | -5.1 |
| 260800 | hypothetical protein | PM - integral | 3 | 5' | -5.1 |
| 300160 | hypothetical protein | NA | 3 | 5' | -3.7 |
| 319720 | hypothetical protein | Nucleus - chromatin | 5 | 5' | -4.49 |
| 207920 | hypothetical protein | Nucleus - chromatin | 2 | 3' | -0.42 |
| 211060 | hypothetical protein | Mitochondrion - membranes | 2 | 3' | -3.26 |
| 221180 | hypothetical protein | Micronemes | 4 | 3' | 0.93 |
| 221220 | hypothetical protein | NA | 2 | 3' | 0.46 |
| 221430 | hypothetical protein | NA | 2 | 3' | -3.38 |
| 222250 | hypothetical protein | NA | 4 | 5' | -4.47 |
| 226340 | hypothetical protein | Nucleolus | 3 | 3' | -2.52 |
| 227880 | hypothetical protein | NA | 3 | 3' | NA |
| 228690 | hypothetical protein | Cytosol | 3 | 3' | -2.11 |
| 235740 | hypothetical protein | Cytosol | 3 | 3' | -2.11 |
| 240270 | hypothetical protein | NA | 3 | 3' | -4.67 |
| 264890 | hypothetical protein | Nucleolus | 5 | 3' | -3.91 |
| 265240 | Hypothetical protein | Nucleus - chromatin | 2 | 3' | -4.34 |
| 267040 | hypothetical protein | Mitrochondrion | 3 | 3' | -4.17 |
| 270720 | Hypothetical protein | NA | 2 | 3' | -4.84 |
| 271290 | hypothetical protein | Nucleus- chromatin, non chromatin | 3 | 3' | -2.02 |
| 277880 | hypothetical protein | Endoplasmic reticulum | 4 | 3' | -0.93 |
| 301250 | hypothetical protein | Nucleus - chromatin | 3 | 3' | -2.04 |
| 308030 | hypothetical protein | NA | 2 | 3' | -4.99 |
| 312670 | hypothetical protein | Nucleus- chromatin, non chromatin | 2 | 3' | -3.04 |
| 313110 | hypothetical protein | N/A | 5 | 3' | -0.4 |
| 313445 | hypothetical protein | NA | 2 | 3' | -1.59 |
| 315460 | hypothetical protein | NA | 3 | 3' | -3.56 |
| 315710 | hypothetical protein | Golgi | 3 | 3' | -0.2 |
| 500346 | hypothetical protein, conserved | NA | 2 | 3' | NA |
